# A Hybrid Nonlinear Greater Cane Rat Algorithm with Teaching–Learning-Based Optimization for Global Optimization and Constrained Engineering Applications

**DOI:** 10.3390/biomimetics11060397

**Published:** 2026-06-04

**Authors:** Jinzhong Zhang, Hongkai Li, Tan Zhang, Zhen He

**Affiliations:** School of Electrical and Photoelectronic Engineering, West Anhui University, Lu’an 237012, China; 42000032@wxc.edu.cn (J.Z.); 42000028@wxc.edu.cn (T.Z.); 2024010821@mail.wxc.edu.cn (Z.H.)

**Keywords:** greater cane rat algorithm, teaching-and-learning-based optimization, standard deviation, teaching and learning, adaptive parameter tuning

## Abstract

The greater cane rat algorithm (GCRA) represents an emerging swarm intelligence paradigm derived from the instinctual survival patterns exhibited by greater cane rats (GCRs), which simulates the typical male-dominated survival patterns of the GCR species, including rainy-season mating and reproduction behaviors, dry-season behavioral differentiation of solitary males and clustered females, and their nonlinear adaptive foraging characteristics. Nevertheless, the original GCRA suffers from inherent defects in complex and high-dimensional optimization scenarios, encompassing premature convergence phenomena, inadequate local exploitation proficiency, constrained convergence precision, and a proneness to stagnation at local optima, which severely restrict its practical engineering application. To address the aforementioned limitations, this work introduces an enhanced hybrid variant of the greater cane rat algorithm, amalgamated with Teaching-and-Learning-Based Optimization (TLBO) and designated as the TLGCRA, incorporating three pivotal targeted innovations. Specifically, the TLGCRA innovatively introduces the two-stage teacher–student interactive learning mechanism of TLBO on the basis of retaining the core evolutionary and behavioral characteristics of the original GCRA, which effectively compensates for the insufficient local disturbance capability of the original algorithm and enriches population diversity to avoid local optimum stagnation. Furthermore, an adaptive parameter tuning strategy is innovatively designed and embedded in the iterative optimization process, which dynamically balances the global exploration and local exploitation capabilities of the algorithm, fundamentally improving the low learning efficiency and weak mining performance of the GCRA. A suite of computational simulations is conducted across 23 canonical benchmark functions and six representative constrained engineering design optimization scenarios. The introduced TLGCRA is benchmarked against the canonical GCRA, LPSO, and ten cutting-edge metaheuristic approaches. Empirical outcomes substantiate that the TLGCRA attains marked performance advantages in terms of convergence velocity, solution precision, and algorithmic resilience. In particular, the optimized design effectively improves the optimal solution precision of the algorithm in complex multimodal function optimization, and the standard deviation of multiple independent runs in six engineering application cases is close to zero, verifying its excellent stability. Statistical verification employing the Friedman test and Wilcoxon signed-rank test additionally corroborates that the TLGCRA exhibits statistically robust and dependable optimization efficacy. In summary, the proposed innovative fusion strategies endow the TLGCRA with stronger environmental adaptability and comprehensive optimization performance, enabling it to realize faster convergence speed and higher computational accuracy, as well as outstanding stability and robustness, thus furnishing a viable resolution framework for intricate constrained engineering optimization challenges.

## 1. Introduction

Since the 21st century, traditional optimization methods built on mathematical derivation have been widely used in engineering design production scheduling and other scenarios. Current swarm intelligence paradigms often struggle to achieve globally optimal outcomes while displaying a pronounced tendency for premature convergence to local optima, particularly when addressing challenging optimization tasks that exhibit high-dimensionality characteristics, pronounced nonlinear dynamics, and strongly coupled variable interactions. Metaheuristic optimization solvers have emerged as pivotal instruments for addressing intricate optimization tasks, owing to their streamlined architecture, adaptable parameter configurations and notable resilience. Swarm intelligence-based methodologies represent a dominant research trajectory within this domain. These approaches maintain a dynamic equilibrium between global exploration and local exploitation by emulating the behavioral characteristics of natural biological communities. These paradigms depart from the rigid mathematical derivations of conventional optimization techniques. These methodologies derive inspiration from natural biological collective behaviors and physical phenomena including bird migration, bee foraging, biological evolution and ant colony searching. Scholars have devised a diverse array of metaheuristic solvers by drawing on the collaborative and adaptive operational mechanisms observed in biological communities. Such solvers aid in resolving various types of complex optimization challenges. Metaheuristic approaches exhibit simplified configurations, adjustable parameter mechanisms, and superior anti-disturbance performance. These methodologies have found extensive utility across diverse interdisciplinary domains including high dimensional engineering optimization, uncertain decision-making and intelligent scheduling. These solvers sustain a dynamic equilibrium between global search and local in-depth exploration. These paradigms compensate for the limitations of conventional optimization techniques, including proneness to local optima and poor adaptability to complex constraints. These methodologies have emerged as core instruments to address contemporary complex practical optimization challenges. Swarm intelligence optimization paradigms constitute a significant subcategory of metaheuristic approaches. These paradigms mimic the collective behaviors of biological communities during foraging and exploratory activities. Numerous experimental studies and real-world deployments have validated that swarm intelligence metaheuristic approaches can be primarily classified into four principal categories. These methodologies are extensively deployed to address nonlinear and high-complexity engineering optimization challenges. Particle swarm optimization (PSO), differential evolution (DE) and covariance matrix adaptation evolution strategy (CMA-ES) serve as quintessential exemplars of metaheuristic approaches. These paradigms have established the theoretical underpinnings for intelligent optimization methodologies. Each of these approaches exhibits inherent limitations. Particle swarm optimization exhibits a tendency toward premature convergence. Differential evolution displays high sensitivity to parameter settings. The covariance matrix adaptation evolution strategy suffers from substantial computational complexity. These inherent limitations hinder their adaptability to the optimization requirements of contemporary complex engineering scenarios.

(1) Biological evolutionary algorithms (BEAs)

Bio-inspired evolutionary algorithms mimic biological heredity, mutation, selection and survival of the fittest to implement optimization through population iteration. They initialize a random solution population, and then adopt crossover to inherit excellent traits and mutation to diversify populations and avoid local optima. Superior individuals are selected iteratively by the fitness function to advance population evolution, until converging to optimal or suboptimal solutions. As population-based global search methods, these algorithms are problem-independent and highly versatile, requiring no gradient information and being insensitive to dimensionality. By balancing global exploration and local exploitation via crossover and mutation, they also effectively prevent premature convergence. Typical algorithms include the Avalanche Algorithm (SAA) [1], Liver Cancer Algorithm (LCA) [2], Coronavirus Mask Protection Algorithm (CMPA) [3], Water Optimization Algorithm (WAO) [4], Poplar Optimization Algorithm (POA) [5], and the Starling Murmuration Optimizer (SMO) [6].

(2) Swarm intelligence algorithms (SIAs)

Swarm intelligence algorithms (SIAs) are an important branch of metaheuristics. They model natural swarm behaviors by simulating local perception, global emergence, feedback regulation, random–deterministic fusion and group collaboration. SIAs mimic the cooperative activities of biological groups, using simple local interactions among individuals to generate global collective intelligence. Through information sharing and dynamic adjustment, they balance solution space exploration and exploitation to converge to the global optimum. Decentralized and collaborative in nature, each individual follows simple local rules, and global optimization is achieved via inter-individual interaction. Independent of the continuity and differentiability of objective functions, SIAs adapt well to complex optimization problems. They are insensitive to initial values, more robust to local optima than single-search methods, and feature excellent stability, wide applicability and parallel computing capabilities. Typical algorithms include the Eel and Grouper Optimization algorithm (EGO) [7], Black-winged Kite Algorithm (BKA) [8], Horned Lizard Optimization Algorithm (HLOA) [9], greater cane rat algorithm (GCRA) [10], Hippopotamus Optimization (HO) [11], Improved Sheep Optimization algorithm (ISO) [12], Walrus Optimizer (WO) [13], and Snow Goose Algorithm (SGO) [14].

(3) Human-inspired algorithms (HBAs)

Human-inspired algorithms (HBAs) are derived from human social behaviors, with logic that closely matches practical decision-making. Free from complex mathematical conversion, they directly reflect real-world collaboration and learning demands and feature low application barriers. Simulating human behaviors of teaching–learning, imitation–innovation and division–cooperation, HBAs realize targeted information interaction. Their efficient collaboration avoids blind search, converges fast to high-quality solutions, and improves optimization accuracy for complex multi-constraint problems. With inherent competition–cooperation and trial-and-error mechanisms, HBAs flexibly regulate search strategies. They effectively balance global exploration and local exploitation, effectively avoiding local optima and premature convergence. HBAs rely on no mathematical properties of objective functions, and are applicable to nonlinear, multi-objective and high-dimensional optimization. They are insensitive to initial values and noise, maintaining stable performance in dynamic scenarios. Moreover, HBAs can be hybridized with swarm intelligence and evolutionary algorithms, and flexibly adjust parameters and collaboration rules with domain knowledge, possessing high flexibility and easy expandability. Typical algorithms include the Information Acquisition Optimizer (IAO) [15], Human Evolution Optimization Algorithm (HEOA) [16], Puma Optimizer (PO) [17], ELK Herd Optimizer (EHO) [18], Memory Backtracking Strategy (MBS) [19], Partial Reinforcement Optimizer (PRO) [20], and Human Memory Optimization Algorithm (HMO) [21].

(4) Physics/Chemistry/Mathematics-inspired algorithms

Physics/chemistry/mathematics-inspired algorithms map optimization problems to physical laws, chemical reactions and mathematical logic. They perform solution searching and optimization by simulating natural operational mechanisms, essentially establishing a mapping from physical, chemical and mathematical rules to solution space search. Relevant discipline principles guide the design of solution update rules. Physics-inspired algorithms simulate energy transfer and gravitational effects to drive particle movement. Chemistry-inspired algorithms mimic chemical bond rearrangement and molecular collision for molecular splitting and synthesis. Mathematics-inspired algorithms generate new search schemes based on probability distribution and logical rules. Candidate solutions are further screened by fitness evaluation to retain high-quality solutions and eliminate inferior ones. Such algorithms balance exploration and exploitation to avoid local optima and search stagnation. Combining deterministic rules with random search, they guarantee optimization reliability while reducing redundant searches. Independent of the mathematical properties of objective functions, they adapt well to complex nonlinear, high-dimensional, multi-objective and multi-constraint problems. Insensitive to initial values and noise interference, they maintain stable optimization performance in dynamic and uncertain environments. Typical algorithms include the wave optics optimization (WOO) [22], Artemisinin Optimization algorithm (AO) [23], Geophysics-based Efficient Optimization Algorithm (FATA) [24], Newton–Raphson-Based Optimization algorithm (NRBO) [25], Fick’s Law Algorithm (FLA) [26], Young’s Double-Slit Experiment Algorithm (YDSE) [27], Lévy Arithmetic Algorithm (LAA) [28], and the Triangular Topology Aggregation Optimizer (TTAO) [29].

The GCRA represents an emerging swarm intelligence optimization approach which imitates the inherent survival activities of cane rats. The algorithm builds nonlinear foraging and survival strategies according to the core behavioral patterns of cane rats. Cane rats spread out for food and reproduce in the rainy season. Female cane rats live in groups and male cane rats live alone in the dry season. The algorithm breaks away from the fixed iteration paradigm of traditional intelligent algorithms. It takes biological habits as the core driving force to promote the whole search process. The algorithm has many natural advantages, such as low computational complexity, no requirement for gradient information and a strong global exploration ability. It possesses a favorable adaptive capacity when handling fundamental optimization tasks. The GCRA exhibits optimization capabilities comparable to conventional metaheuristic methods in initial observation. The typical comparative algorithms cover PSO, DE and CMA-ES. However, essential differences exist in the underlying iteration logic search framework and evolution driving mode between the GCRA and these algorithms. PSO adopts the velocity position iteration framework. The update of particle position depends entirely on the two-way guidance of individual optimum and global optimum. The velocity component governs search orientation and moving step length. This algorithm tends to be greatly influenced by outlier individuals. It lacks valid strategies to escape once trapped into local optimal solutions. The algorithm is a velocity iteration method guided by extreme values in nature. DE drives population evolution through three manually designed operators. The three operators include differential mutation, crossover and selection. The optimization performance of the algorithm relies heavily on manual adjustment of control parameters. The control parameters include the scaling factor and crossover rate. The global search of the algorithm has strong randomness but low convergence efficiency. The algorithm has high parameter sensitivity. It can be classified as an optimization algorithm based on artificial evolution operators. The CMA-ES realizes optimization through adaptive adjustment of the covariance matrix and Gaussian sampling. The iteration process involves matrix eigen decomposition. The algorithm has extremely high computational complexity. Computational cost increases exponentially with the rise in problem dimensions. The algorithm is only suitable for low-dimensional optimization scenarios with high precision requirements. It is difficult to meet the needs of large-scale engineering real-time optimization. In contrast, the GCRA completely abandons the velocity position iteration framework. It takes the real biological habits of cane rats as the core driving force. The algorithm switches naturally between exploration and development stages by judging seasonal changes in rainy and dry seasons. Population renewal is realized through the territory guidance of dominant male rats and the reproductive behavior of female rats. The whole search process conforms to the natural survival logic of creatures instead of artificial velocity iteration rules. The GCRA has no manual operators such as mutation and crossover. It completes iteration only through natural behavior rules. These behaviors include foraging, reproduction and territory patrol. The algorithm has few parameters and no need for complex adjustment. The algorithm dynamically adjusts the search step size with nonlinear strategies. It can balance global exploration and local development efficiently. The GCRA is an adaptive optimization algorithm driven purely by biological behaviors. In addition, the GCRA involves no complex mathematical operations such as matrix operations and Gaussian sampling. The iteration logic is simple and straightforward. The algorithm has low computational cost. It can still maintain efficient search in high-dimensional scenarios. The algorithm is especially suitable for time-delay-sensitive application scenarios. These scenarios include embedded real-time optimization and large-scale engineering scheduling. The GCRA has no homology with the three classic algorithms in the underlying search paradigm.

The nonlinear cane rat algorithm integrated with sine and cosine functions (SCGCRA) proposed in existing studies is a typical hybrid algorithm with external strategy splicing [30]. Its core idea is to retain the complete iterative framework of the traditional GCRA, merely embed the Sine Cosine Algorithm (SCA) as an external mathematical operator, and partially revise the position update rules. This algorithm fully adopts the fixed two-stage iterative paradigm of rainy and dry seasons of the GCRA, taking seasonal judgment as the switching criterion in the balance of global search and local exploitation. Population update still relies on the guidance of dominant male rats. The nonlinear fluctuation formulas of sine and cosine from the SCA are only introduced in the foraging position update process. The periodic oscillation of trigonometric functions replaces the original linear search step size, and the nonlinear attenuation coefficient of the SCA realizes the flexible modification of global search and local exploitation. In terms of integration essence, the SCGCRA is a surface-level integration driven by mathematics. It only optimizes the search trajectory by virtue of the nonlinear characteristics of trigonometric functions, without changing the original population interaction mode, iterative logic and biologically driven core of the GCRA, or reconstructing the individual information transmission mechanism. This results in obvious drawbacks of the SCGCRA. Extra calibration of the sine- and cosine-related parameters of the SCA is required, bringing high parameter sensitivity. Nonlinear oscillation lacks directional guidance, leading to limited solution accuracy. Trigonometric operations bring additional computational costs. The fixed two-stage iteration mode lacks flexibility with no population information interaction. The search solely depends on trigonometric oscillation. In the process of handling high-dimensional multimodal problems, exploration and exploitation fail to coordinate effectively, and the algorithm tends to fall into local optimum prematurely. Based on the comparison of advantages and disadvantages of the above algorithms, this paper integrates the teaching–learning optimization mechanism into the GCRA to fabricate a mixed optimization strategy with superior performance.

Three representative improved teaching–learning optimization algorithms published in recent international journals provide important references for the design and development of the teaching–learning-based greater cane rat algorithm. The Golden Ratio Opposition Enhanced Teaching–Learning Optimization (GRO-TLBO) approach developed in 2024 embeds the learning strategy based on golden ratio opposition property into the original TLBO framework [31]. It effectively enriches population diversity and speeds up convergence speed. This method has been verified in the damage detection of laminated composite beams. Developed in 2026, the Knowledge-Guided Teaching–Learning Optimization (KG-TLBO) approach embeds discrete crossover with a topology retention property and critical path-dependent local searching tactics [32]. This approach achieves high efficiency in handling discrete optimization challenges for flexible job shop scheduling. The Enhanced Educational Competition Optimizer (EECO) proposed in 2025 optimizes the three-phase learning architecture and incorporates a novel population regeneration strategy [33]. It alleviates the problem of unstable convergence in the later stage of algorithm iteration. All these improved algorithms retain the fixed iteration structure of traditional teaching–learning optimization algorithms. They fail to realize deep integration with biological inspiration mechanisms. Certain limitations still exist in complex optimization scenarios.

The core merits of the TLGCRA reside in the integration of the complementary strengths of the GCRA and TLBO, while remediating the limitations of conventional algorithms via dynamic parameter tuning and chaotic mutation mechanisms The TLGCRA proposed in this paper is a hybrid algorithm with endogenous mechanism reconstruction. It does not simply superimpose operators. It deeply reconstructs the underlying iteration logic and population interaction rules on the basis of the biological habit framework of the GCRA. This algorithm retains the core driving nature of the GCRA, which is based on natural survival behaviors. It completely abandons the constraint of fixed two-stage iteration. It dynamically integrates the two-stage mechanism of TLBO with the behavioral habits of cane rats. Specifically, the teacher stage of TLBO is used to replace the territory guidance of single dominant male cane rats in the GCRA. The global optimal individual is taken as the teacher to implement directional guidance. This strengthens the transmission efficiency of high-quality solutions. The student mutual learning stage of TLBO is adopted to build a multi-individual interactive learning architecture. This breaks the defect of the single information interaction mode in the traditional GCRA. It realizes the sharing of multiple pieces of information within the population. The algorithm abandons the update rule by relying on population mean at the same time. It realizes the dynamic and autonomous switching of search strategies combined with adaptive weight adjustment. The TLGCRA is essentially a deep fusion driven by both biological behavior and intelligent learning. It reconstructs the population evolution logic from the endogenous level. It is not a simple implantation of external mathematical operators. Compared with existing algorithms, the TLGCRA has certain advantages. Its research achievements and strengths are as follows: (1) Realizing diverse information interaction through teacher guidance and peer learning among students can sustain population diversity in the long term and effectively restrain premature convergence. (2) Utilizing dynamic autonomous switching tactics can make real-time modification on search modes based on iterative evolution and population distribution characteristics, greatly improving the convergence stability in high-dimensional scenarios. (3) It has no complex mathematical operations and has concise iterative logic and lower computational complexity. It is more applicable to delay-sensitive scenarios such as embedded real-time optimization and large-scale scheduling. (4) It is able to achieve targeted transmission of high-quality solutions by relying on teaching mechanisms with enhanced local exploitation capabilities. In the meantime, this method shows remarkable performance in avoiding local extremum, relying on the interactive behaviors of multiple agents, thus reaching a more ideal equilibrium of global exploration and local exploitation. (5) This solver exhibits low parameter complexity and simple configuration and requires no complex tuning, boasting superior stability and generalization abilities. (6) Adopting a hybrid architecture improves the efficiency of population information interaction, avoids blind search issues caused by scarce candidate solutions in the high-dimensional solution space, and mitigates the negative impacts of the curse of dimensionality. In tests of hundred-dimensional functions, the algorithm presents a low average fitness value and a small standard deviation, with stability performance noticeably superior to comparative algorithms such as the cane rat optimization algorithm and gravitational-force-oriented search strategy. (7) The TLGCRA is contrasted with a range of state-of-the-art algorithms, including recently released, widely cited and high-efficiency ones such as LPSO, SBKA, ESGJO, MSSCSO, PO, WSA, HLOA, ECO, IAO, AO, HEOA, and NRBO, as well as the basic version of the GCRA. (8) Via experimental simulations and outcome evaluation, the TLGCRA is validated and assessed on 23 benchmark functions and six practical engineering design tasks.

The remainder of this paper is structured as follows. Section 2 details the fundamental mechanisms and overall framework of the GCRA. Section 3 comprehensively illustrates the design scheme and enhancement strategies of the developed TLGCRA. Section 4 conducts benchmark function simulations and discusses the corresponding experimental results. Section 5 validates the practical feasibility of the TLGCRA through typical engineering design cases. Section 6 concludes the overall research work and outlines prospective future research directions.

## 2. Greater Cane Rat Algorithm (GCRA)

The greater cane rat algorithm (GCRA) is an emerging metaheuristic optimization approach constructed based on the authentic biological habits and behavioral traits of the greater cane rat. Its core principle lies in emulating the intelligent foraging behaviors and collective cooperation mechanisms of the target species. Digital simulations of territory exploration, path tracking and reproduction strategies help the algorithm evolve into a high-performance optimizer capable of effectively addressing diverse complex optimization tasks. The algorithm exhibits outstanding capabilities in both global exploration of uncharted solution spaces and precise local excavation of high-quality candidate solutions. The greater cane rat is a typical nocturnal rodent that mainly inhabits humid and near-water areas such as swamps, river banks and farmlands. It feeds on sugarcane and herbaceous plants as its main food sources. The algorithm accurately restores the core behavioral characteristics of the species. Individuals spread out for food during non-breeding periods and gather for reproduction during breeding periods. This behavior pattern changing dynamically with seasons provides the core basis for the algorithm to achieve a dynamic balance between global exploration and local optimization. Moreover, greater cane rats in natural habitats typically forage along predefined trails formed by prostrate herbaceous vegetation. This behavioral feature is also integrated into the design of path tracking and optimization-seeking mechanisms of the algorithm. As a new-generation swarm intelligence optimization paradigm, the GCRA possesses fundamentally distinct population iteration logic and evolution mechanisms compared with conventional metaheuristic algorithms such as PSO, DE and CMA-ES. These classic algorithms have been widely used in various optimization scenarios, but all have inherent defects that are difficult to avoid. The GCRA has delivered innovative advancements at the fundamental operational mechanism level. The proposed method fully discards the velocity and position update framework of PSO, the differential mutation scheme of DE and the Gaussian distribution sampling pattern of the CMA-ES. It drives population iteration and updates by relying on independent evolution rules. The algorithm integrates individual neighborhood features and adaptive weight strategies. It maintains a dynamic balance between global exploration and local exploitation processes without overreliance on global optimal solutions to steer the optimization procedure. The algorithm also boasts benefits including low computational complexity, robust capability to escape local optima and stable convergence performance. Its operation mechanism has no homology with traditional classic algorithms. It can effectively mitigate the prevalent drawbacks of traditional algorithms such as premature convergence tendency, slow convergence rate and high computational overhead.

Overall, the GCRA addresses the practical limitations of conventional metaheuristic solvers. It presents new research perspectives and innovative directions in the advancement of intelligent optimization methodologies. The algorithm further refines the theoretical framework of metaheuristic approaches. It delivers innovative schemes and practical strategies to tackle diverse real-world intricate engineering optimization challenges. The algorithm possesses important theoretical research value and engineering application value.

### 2.1. Population Initialization

The matrix of the randomly generated initial population is defined as follows:

(1) $$X=\left[\begin{array}{ccccc} x_{1,1} & x_{1,2} & \ldots & x_{1,d-1} & x_{1,d}\\ x_{2,1} & x_{2,2} & \ldots & x_{2,d-1} & x_{2,d}\\ \vdots & \vdots & x_{i,j} & \vdots & \vdots \\ x_{n,1} & x_{n,2} & \ldots & x_{n,d-1} & x_{n,d} \end{array}\right]$$

where *X* represents the GCR population, $x_{i,j}$ represents the jth position in the dimension, n represents the population size, and d stands for the dimensionality of the problem. $x_{i,j}$ is calculated as follows:

(2) $$x_{i,j}=rand\times (UB_{j}-LB_{j})+LB_{j}$$

where rand∈[0,1], UB and LB represent the upper and lower bounds.

The dominant giant cane rat (GCR) individual $x_{k,j}$ is regarded as the most adaptive member of the colony, capable of directing the collective toward familiar foraging grounds and habitats, revising individual positions, and warding off unguided random searches. Variable ρ=0.5, by contrast, functions as a seasonal discriminator that defines the onset of the rainy season, a mechanism employed to dynamically toggle between the algorithm’s global exploration and local exploitation stages.

(3) $$x_{i,j}^{new}=0.7\times \frac{\left(x_{i,j}+x_{k,j}\right)}{2}$$

where $x_{i,j}^{new}$ represents the updated position and $x_{i,j}$ represents the current.

### 2.2. Exploration

The GCRs establish dens or shallow burrow refuges that are dispersed throughout their territorial habitats—including swamps, riverbanks, and cultivated lands. This distributed shelter system facilitates dispersed migratory foraging behavior, while trail markers are generated via territorial patrolling and route tracing mechanisms. The optimum position of the leading male GCR is defined as the food-source trajectory, and the remainder of the population adheres to this path while modifying their respective positions. This behavioral tactic empowers the colony to explore distinct areas, extend the bounds of the solution space, avert search stagnation, and generate a diverse array of candidate solutions. The position is computed as follows:

(4) $$x_{i,j}^{new}=x_{i,j}+C\times (x_{k,j}-r\times x_{i,j})$$

(5) $$x_{i}=\left\{\begin{array}{c} x_{i,j}+C\times (x_{i,j}-\alpha \times x_{k,j}),\;F_{i}^{new}<F_{i}\\ x_{i,j}+C\times (x_{m,j}-\beta \times x_{k,j}),\;\mathrm{otherwise} \end{array}\right.$$

Herein, $x_{i}$ signifies the updated position of an individual ith GCR, $x_{i,j}^{new}$ indicates the coordinate associated with the jth dimension, and $x_{i,j}$ stands for the GCR’s present position. $x_{k,j}$ represents the position of the leading male GCR, whereas $F_{xk}$ signifies the fitness value of $x_{k,j}$ and $F_{xi}$ denotes the individual’s present fitness. C∈[0,1] depicts the dispersed distribution of food resources and refuges throughout the habitat. γ mimics the influence of rich and diverse food resources, thereby boosting the algorithm’s exploitation performance. α simulates the scenario of declining food supplies, which compels the GCR population to explore newly emerged food sources and shelters. β drives the GCR individuals to migrate to alternative resource-rich habitats inside their respective breeding territories. γ, α, β are calculated as follows:

(6) $$r=F_{xk}-t\times (\frac{F_{xk}}{T})$$

(7) α=2×r×rand−r

(8) β=2×r×μ−r

where t represents the current iterative step and T represents the maximum iterative cycle.

### 2.3. Exploitation

During the rainy season, sexually active male GCRs depart from their colonies to conduct in-depth foraging in regions with abundant food supplies. The GCRA leverages the stochastic selection of female GCRs to emulate the precise local utilization of superior-quality regions through procreative behaviors, and this mechanism strengthens the precision of local search processes and improves the overall quality of solutions. The location is calculated as follows:

(9) $$x_{i,j}^{new}=x_{i,j}+C\times (x_{k,j}-\mu \times x_{m,j})$$

where $x_{m,j}$ signifies the female GCR’s position and μ∈[1,4] mimics the number of offspring generated by each female GCR.

Algorithm 1 illustrates the algorithmic pseudocode of the GCRA.

**Algorithm 1** GCRA

**Begin**

**Step 1.** Initialize the GCR colony $X_{i}(i=1,2,\ldots ,n)$

**Step 2.** Evaluate the fitness of GCRs, update the global best solution (Gbest)
    Select the most adaptive GCR as the leading male $x_{k}$
    Update the remaining GCRs derived from $x_{k}$ via Equation (3)
**Step 3. while** t<T do
    **for** all GCRs
        Renovate ρ, r, α, β, C, μ
        **if** rand<ρ
        **Exploration**
        Update GCRs positions via Equation (4)
        **else**
        **Exploitation**
        Update GCRs positions via Equation (9)
        **end if**
    **end for**
    Verify whether any solution exceeds the search boundary and correct it
    Evaluate the fitness of GCRs based on updated positions
    Update GCRs positions via Equation (5)
    Update Gbest and select a new leading male $x_{k}$
    t=t+1
    **end while**
    **Return** Gbest

**End**



### 2.4. Nonlinear GCRA

The progressively diminishing attenuation pattern of the parameter amplitude is calibrated against the inherent structural architecture of the algorithm. The nonlinear control scheme demonstrates a powerful robust disturbance rejection capability and superior nonlinear processing performance. This design not only guarantees control precision and operational robustness, but also boosts the global exploration efficiency and local exploitation capability. It further reinforces the algorithm’s adaptiveness and practical realizability, promoting dynamic parameter adjustment alongside the fidelity of the ultimate optimal solutions. The position revisions are calculated as follows:

(10) $$W=2\cdot e^{-{\left(\frac{8t}{T}\right)}^{2}}$$

(11) $$x_{i,j}^{new}=W\times x_{i,j}+C\times (x_{k,j}-r\times x_{i,j})$$

(12) $$x_{i}=\left\{\begin{array}{c} W\times x_{i,j}+C\times (x_{i,j}-\alpha \times x_{k,j}),\;F_{i}^{new}<F_{i}\\ W\times x_{i,j}+C\times (x_{m,j}-\beta \times x_{k,j}),\;\mathrm{otherwise} \end{array}\right.$$

(13) $$x_{i,j}^{new}=W\times x_{i,j}+C\times (x_{k,j}-\mu \times x_{m,j})$$

where t represents the present iteration and T represents the upper bound of iterations.

## 3. Teaching–Learning-Based Greater Cane Rat Algorithm (TLGCRA)

Most existing teaching–learning-based algorithms adhere to a fixed two-stage iterative structure and a single teacher–student learning mode. Their improvement methods mainly rely on parameter tuning and simple strategy integration, making it difficult to break through the inherent limitations of the algorithms at the fundamental level. The TLGCRA integrates the complementary advantages of the GCRA and TLBO. Its unique interactive learning paradigm can effectively compensate for the deficiencies of the GCRA in population information communication and parameter self-adaptation capability. It breaks away from the constraints of the traditional teaching–learning framework, reconstructs autonomous neighborhood learning and adaptive evolution mechanisms, and abandons rigid teacher–student pairing and mean-guided update modes. Expanding from the teacher-to-student teaching mode to the student-to-student teaching mode, it is no longer limited to a single mode. It achieves differentiated innovation at the level of underlying iterative logic and population interaction rules, effectively solving the problems of traditional teaching–learning algorithms, such as monotonous learning modes, premature convergence, and poor search coordination. It exhibits distinct innovation and performance advantages compared with similar teaching–learning-based methods. Meanwhile, the proposed algorithm alleviates the limitations of traditional algorithms through adaptive parameters and chaotic mutation operators, showing remarkable superiority in optimization accuracy and robustness. This algorithm can effectively avoid local optimal solutions and thereby facilitate the acquisition of high-quality optimal solutions.

### 3.1. Teaching-and-Learning-Based Optimization (TLBO)

For the teaching phase, TLBO emulates the knowledge transmission paradigm from a distinguished teacher to the entire class, where the global optimum of the present population is defined as the teacher $X_{\mathrm{teacher}}$. This phase prioritizes local exploitation by contracting the search space toward the optimal region, while embedding stochasticity to prevent population homogeneity and sustain evolutionary diversity. The core mechanism hinges on quantifying the knowledge gap between the teacher solution and the population mean solution $X_{\mathrm{mean}}$, which drives individual updates toward promising search areas. The teaching factor $T_{f}$, a binary random variable taking values in [1,2], further introduces adaptive disturbance to attain a dynamic equilibrium between the convergence rate and the robustness of the global search. The associated position revision equation is presented as follows:

(14) $$D_{difference}=\mathrm{rand}\left(0,1\right)\left(X_{\mathrm{teacher}}-T_{f}X_{\mathrm{mean}}\right)$$

(15) $$T_{f}=\mathrm{round}\left(1+\mathrm{rand}\left(0,1\right)\right)$$

(16) $$X_{new}^{i}=X_{old}^{i}+D_{difference}$$

where $X_{\mathrm{teacher}}$ represents the optimal individual of the population objective function, $X_{\mathrm{mean}}$ signifies the mean performance level of the cohort, $T_{f}$ indicates the instructional factor, and i denotes that the ith student updates its position according to the formula.

This study discards the unidirectional and monotonous individual update paradigm adopted in the learning phase of the classical teaching–learning-based optimization (TLBO) algorithm. Targeting two critical drawbacks of the original formula, namely the absence of interactive differential learning among individuals and the non-uniform dimensional matching of parametric terms, an innovative updating paradigm centered on dual-individual differential mutual learning is proposed in this work. Different from the conventional TLBO strategy that restricts individual learning to one-way iteration toward the optimal population agent, the developed paradigm establishes a differential learning framework based on three mutually independent and heterogeneous individuals in the population. The dimensional deviations between paired individuals are adopted to construct adaptive learning perturbation terms, while normalization processing is implemented for both learning coefficients and differential vectors. Such operation achieves rigorous dimensional uniformity of the entire iterative formula and restores the essential interactive learning principle among population individuals. In the proposed paradigm, $x_{i,j}^{new}$ denotes the initial position vector of the individual to be optimized, which serves as the basic iterative updating benchmark. This fundamental variable can efficiently retain the inherent feature information of individuals and prevent the loss of elite population information caused by excessive perturbation during iteration. $r_{1}$ and $r_{2}$ represent the normalized differential learning coefficient, which substitutes the unnormalized random perturbation parameters used in traditional TLBO variants. This improvement thoroughly resolves the dimensional contradiction arising from the mixed superposition of constant parameters, perturbation terms and variable terms, enabling all iterative step sizes to possess standardized and physically consistent characteristics. $\left(x_{m,j}-x_{i,j}\right)$, $\left(x_{n,j}-x_{i,j}\right)$ refers to the dual differential learning term, which constructs differential vectors via screening mutually independent heterogeneous individuals. This term precisely depicts the internal interactive learning behavior of the population and allows the target individual to capture dimensional feature deviations from two different peer agents, thereby overcoming the core limitation of traditional TLBO that only supports unidirectional learning without individual differential interaction. Taking heterogeneous individual differential vectors as the core driving factor of iterative renewal, the improved updating strategy enables individual optimization to fully refer to multi-agent feature discrepancies within the population, which strictly implements the original design intention of peer interaction and mutual learning of the TLBO algorithm. In exploitation of the TLGCRA, the location is calculated as follows:

(17) $$x_{i,j}^{new}=x_{i,j}+r_{1}(x_{m,j}-x_{i,j})+r_{2}(x_{n,j}-x_{i,j})$$

where $x_{i,j}^{new}$ denotes the optimized position coordinate of the i individual in the j dimension after one round of differential learning iteration, and it serves as the core iterative output of the update rule in the improved learning stage. $x_{i,j}$ represents the initial coordinate of the i individual in the j dimensional search space of the population. $r_{1}$ and $r_{1}$ refer to the normalized differential learning factor, which is an independent uniformly distributed random scalar, rand∈[0,1]; $x_{m,j}$, $x_{n,j}$ represent the dimensional coordinates of two different individuals randomly selected from the population; and the subscripts m, n, and i denote the independent population number, and m≠n≠i.

### 3.2. TLGCRA

While the conventional Teaching–learning-based optimization (TLBO) algorithm boasts a straightforward structure, minimal parameters and strong robustness, it suffers from obvious mechanical defects. In the core peer learning phase, individuals in the population complete the information interaction and iterative updates by merely relying on peers at the same level. Restricted by the limited search depth and solution accuracy of peer individuals, the algorithm is highly prone to insufficient knowledge learning accuracy and slow iterative convergence speed. Meanwhile, mutual learning, collaboration and adaptation among individuals consume a large number of iterations, further slowing down the overall optimization efficiency. In addition, the group collaboration mode tends to make individuals overly dependent on peer information, weakening their ability of independent search and independent thinking, and reducing the autonomous optimization-seeking capability of population individuals. Furthermore, due to differences in the search directions and information characteristics of peer individuals, problems such as opinion conflicts, poor collaboration and information confusion frequently occur during the iteration process. This leads to a notable reduction in the algorithm’s optimization performance during later iterations, hindering its ability to rapidly converge to superior optimal solutions and severely limiting its optimization capacity when dealing with high-dimensional complex optimization tasks. Therefore, rather than directly utilizing the standard TLBO algorithm, this work deeply integrates its learning paradigm with the two-stage adaptive search mechanism from the GCRA, and introduces dynamic parameter tuning and chaos-based mutation strategies. While retaining the advantages of the baseline GCRA, such as its compact structure and limited number of adjustable parameters, the enhanced algorithm eliminates aimless search behavior through its guidance stage and quickly drives the population toward the global optimal solution, thus substantially accelerating the convergence speed. Meanwhile, the global foraging exploration ability of the GCRA offsets the drawbacks of TLBO, such as inadequate search breadth in peer learning and excessive individual reliance. It avoids group collaboration conflicts by virtue of the independent partition search feature, and corrects the problems of low peer learning accuracy, sluggish convergence rates and extended adaptation durations through chaotic mutation perturbation. The enhanced algorithm effectively overcomes the intrinsic drawbacks of the original TLBO algorithm and achieves a balanced coordination between global exploration and local exploitation.

Algorithm 2 illustrates the algorithmic pseudocode of the TLGCRA.

**Algorithm 2** TLGCRA

**Begin**

**Step 1.** Initialize the GCRs colony $X_{i}(i=1,2,\ldots ,n)$
**Step 2.** Evaluate the fitness of GCRs, update the global optimal solution (Gbest)
    Select the most adaptive GCR as the leading individual $x_{k}$
    Update the remaining GCRs derived from $x_{k}$ via Equation (3)
**Step 3. while** t<T do
     **for** all GCRs
      Renovate ρ, r, α, β, C, μ
      **if** rand<ρ
       **Exploration**
       The nonlinear control strategy is introduced into exploration of GCRA
       Renovate GCRs positions via Equation (4)
      **else**
       **Exploitation**
       The nonlinear control strategy is introduced into exploration of GCRA
       Renovate GCRs positions via Equation (9)
      **end if**
     **end for**
     Affirm whether any solution has overflowed the search interval and revise it
     Estimate the fitness of GCRs stem from a renewed location
     Update the positions of GCRs via Equation (5)
     Update Gbest and select the updated leading individual $x_{k}$
     The reaching-learning-based optimization is introduced into GCRA
     Update the positions of GCRs via Equations (16) and (17)
     t=t+1
    **end while**
    **Return** Gbest

**End**



As an endogenous reconstructed hybrid algorithm that deeply integrates the biological habits of cane rats and the teaching–learning-based optimization mechanism, the TLGCRA possesses distinctive innovative features and superior performance compared with three classic intelligent optimization algorithms, including PSO, DE and CMA-ES, which are elaborated as follows: (1) It completely breaks away from the velocity–position iteration framework of PSO by relying on extremum guidance, fundamentally avoiding interference from extreme individuals and the drawback of premature convergence. (2) It overcomes the limitations of DE such as the requirement for precise manual calibration of control parameters, low convergence efficiency and high parameter sensitivity. (3) It eliminates the problems of extremely high computational complexity and poor adaptability to high-dimensional scenarios caused by matrix eigenvalue decomposition and Gaussian sampling in the CMA-ES. (4) Driven mainly by the natural survival behaviors of cane rats and organically combined with the teaching and interaction mechanism of TLBO, the TLGCRA reconstructs the population evolution logic and information interaction mode through targeted guidance from teachers and diversified mutual learning among students. It adopts a dynamic autonomous switching strategy instead of fixed iteration patterns, which can sustain population diversity for a long time and effectively balance global exploration and local exploitation capabilities. (5) The algorithm involves no complex mathematical operations, features lower computational cost and fewer parameters without complicated tuning. It maintains excellent convergence stability and optimization accuracy even in high-dimensional multimodal optimization scenarios, making it more applicable to latency-sensitive practical engineering applications such as embedded real-time optimization and large-scale engineering scheduling. As shown in Table 1, a comparative analysis of the TLGCRA with PSO, DE, CMA-ES and the GCRA from four evaluation dimensions demonstrates that the TLGCRA presents advantages.

## 4. Numerical Simulation Experiments and Performance Analysis for Solving Benchmark Test Functions

### 4.1. Experimental Setup

The experimental testbed is built upon a 64-bit Windows 11 operating system. It is equipped with an 11th-generation Intel Core i7-11800HX processor operating at 2.30 GHz, 4 TB of storage capacity, a 16 GB dedicated graphics unit, and 16 GB of main memory. All competing methods are implemented in MATLAB R2024b.

### 4.2. Benchmark Functions

The TLGCRA employs unimodal functions $\left(f_{1}-f_{7}\right)$, multimodal functions $\left(f_{8}-f_{12}\right)$, and fixed-dimension multimodal functions $\left(f_{13}-f_{23}\right)$ to validate reliability and practicality. Table 2 outlines the benchmark functions.

### 4.3. Parameter Settings

To fully demonstrate the practical effectiveness and applicable scenarios of the TLGCRA, this paper selects 12 classic and improved algorithms (including LPSO, SBKA, ESGJO, RLWOA, MSSCSO, PO, NRBO, EGO, AO, FATA, HEOA, and GCRA) as benchmark algorithms to design and conduct comparative experiments. All experimental parameters are extracted from the original studies of each algorithm, integrating multi-dimensional bases such as previous empirical summaries, theoretical derivation and analysis, adaptive adjustment mechanisms, and hybrid optimization strategies. They are typical values with both representativeness and reliability.

LPSO: precarious value $r_{1}\in \left[0,1\right]$, precarious value $r_{2}\in \left[0,1\right]$, precarious value $\omega \in \left[0.4,0.9\right]$, fixed value β=10, fixed value m=0.8, fixed value $C_{1}=2.05$, fixed value $C_{2}=2.05$.

SBKA: precarious value rand∈[0,1], fixed value p=0.9, precarious value r∈[0,1], Cauchy mutation C∈(0,1), fixed value δ=1, fixed value μ=0.

ESGJO: precarious value rand∈[0,1], fixed value α=0.05, fixed value pi=3.14.

RLWOA: precarious value rand(1,D)∈[0,1], precarious value F∈[0,2], fixed value β=1.5, precarious value $r\in [1,N_{p}]$, precarious value $j_{rand}\in [1,D]$, precarious value CR∈[0,1], precarious value A∈[1,−1], precarious value $\vec{r}\in [0,1]$.

MSSCSO: precarious value $R\in \left[-2r_{G},2r_{G}\right]$, precarious value $\theta \in \left[0,360\right]$, fixed value $s_{M}=2$.

PO: fixed value ${\mathrm{PF}}_{1}=0.5$, fixed value ${\mathrm{PF}}_{2}=0.5$, fixed value ${\mathrm{PF}}_{3}=0.5$, fixed value U=0.2, fixed value L=0.9, fixed value α=2.

NRBO: fixed factor DF=0.6, precarious value r∈(0,1), precarious value δ∈(−1,1), precarious value a∈(0,1), precarious value b∈(0,1).

EGO: precarious value $rand\in \left[0,1\right]$, precarious value $r_{1}\in \left[0,1\right]$, precarious value $r_{2}\in \left[0,1\right]$, precarious value $c_{1}\in \left[-a,a\right]$, precarious value $c_{2}\in \left[0,2\right]$.

AO: precarious value $R\in \left[0,1\right]$, precarious value $r_{1}\in \left[0,1\right]$, precarious value $d\in \left[0.1,0.6\right]$.

FATA: precarious value i∈[1,n], precarious value θ∈[0,1], precarious value α∈[0,1], precarious value $Para_{1}\;\in [0,1]$, precarious value $Para_{2}\in [0,1]$.

HEOA: precarious value $rand\in \left[0,1\right]$, precarious value R∈[0,1], fixed value A=0.5, precarious value t.Rd∈[1,dim].

GCRA: precarious value rand∈(0,1), fixed value ρ=0.5, precarious value μ∈[1,4].

TLGCRA: precarious value rand∈(0,1), fixed value ρ=0.5, precarious value μ∈[1,4].

### 4.4. Numerical Simulation Experiments and Performance Evaluation

To guarantee the objectivity and thoroughness of the performance assessment, the present work adopts consistent experimental parameter settings for all compared algorithms: a population count of 50, a maximum number of iterations set to 1000, and 30 independent trials executed for each algorithm. This experimental configuration facilitates a side-by-side comparative investigation of the convergence characteristics and solution accuracy across different algorithms. The comprehensive comparative findings regarding the benchmark test functions are documented in Table 3.

To tackle benchmark optimization tasks, targeted enhancements were integrated into the TLGCRA framework. The core objectives include addressing the baseline GCRA’s limitations, such as insufficient local exploitation efficiency and vulnerability to local optima traps, boosting the algorithm’s learning and operational capabilities, elevating exploration effectiveness and solution quality and enhancing its overall stability and resilience. The interactive learning paradigm effectively mitigates the GCRA’s deficiencies in population information sharing and parameter adaptability, reducing parameter sensitivity and outcome volatility, while enabling more accurate identification of the global optimum or high-quality near-optimal states. Experimental analyses based on best, worst, mean and standard deviation metrics comprehensively validate the algorithm’s fundamental properties and reveal its reliability and practical applicability across diverse scenarios. As the best solution value converges toward the theoretical global optimum, it indicates an improved capability to uncover high-quality solutions and enhanced performance in escaping local optima. The proximity between the best value and the theoretical global optimum directly determines whether the algorithm can achieve high-quality results and avoid local optimum traps the closer the value is to the theoretical optimum the greater the algorithm’s optimization potential and the stronger its performance in overcoming local stagnation. A rapid decrease followed by stabilization of the best value during iterations demonstrates the algorithm’s fast convergence rate and high computational accuracy. The worst value corresponds to the objective fitness of the poorest solution across multiple independent trials, where each individual’s fitness is computed using the predefined fitness function. This metric assesses the algorithm’s robustness under varying conditions, such as changing initial populations, and evaluates solution stability amid input data variations, thereby reflecting performance in extreme cases. Across repeated trials, the smaller the gap between the best and worst solution fitness values, the lower the algorithm’s sensitivity to initial population distribution parameter perturbations, complex solution space structures, and stochastic factors, and the stronger its robustness. If the worst-case fitness of the enhanced algorithm outperforms that of the baseline, it confirms that the proposed modifications effectively suppress suboptimal solutions, reduce the likelihood of infeasible results and prevent severe local convergence. The mean value captures the average performance of the optimization algorithm across multiple runs. The acceptable range of the mean value varies depending on the optimization task category; typically, a lower objective function value indicates superior solution quality. The mean of the objective function values can serve as the final metric for cross-method comparison, thereby assessing the merits and drawbacks of the algorithm’s overall average efficacy. The proximity between the mean value and the theoretical global optimum can gauge the overall search efficacy of the algorithm. If the mean value approximates the theoretical global optimum more closely, it demonstrates that the algorithm can locate high-quality solutions more effectively with a proper overall search orientation, and otherwise, issues like inadequate search efficiency may arise. If the mean values are consistent across multiple trials and the solution distribution span is broad and even, it reveals that the algorithm can identify solutions of comparable quality within an extensive search domain, exhibiting favorable uniformity in solution distribution, which aids in preventing the algorithm from becoming trapped in local optima. The standard deviation quantifies the extent of dispersion of objective fitness values with respect to the mean value and is employed to assess algorithm performance. A lower standard deviation signifies that the outcomes acquired from each trial of the algorithm are nearer to the mean value, with minor variations in convergence outcomes, high algorithm stability, and robust repeatability. Conversely, a higher standard deviation suggests substantial result variations, elevated algorithm sensitivity to stochastic factors, and comparatively low reliability.

The solution domain of a unimodal function possesses a single global extremum and no additional local extrema. Figuratively speaking, its graph is like an isolated peak; starting from any point, searching along the direction of the function value change will eventually lead to the only peak, without the situation of straying into a small hill. At the same time, a unimodal function is monotonic (showing a monotonically increasing or decreasing trend on both sides of the extremum), which allows the algorithm to clearly judge which direction to search to get closer to the optimal solution. This phenomenon mainly stems from the fact that unimodal test functions contain no local extrema and hold obvious monotonic characteristics. These properties help optimization methods rapidly shrink the searching domain. Algorithms do not have to consume computational resources to differentiate the present search position between the local optimum and global optimum. They only need to run iterations along gradient trends and follow the changing rules of function values to gradually converge to the global optimal solution. Useless searching actions are substantially cut down during the whole process. The search orientation becomes more accurate, which keeps the algorithm from following redundant paths. These benchmarks focus on testing the ability to draw close to optimal solutions at high speed and high accuracy. The results can further assess the algorithm’s local exploitation accuracy, convergence efficiency and compatibility with basic solution spaces. For $f_{1}$, $f_{2}$, and $f_{4}$, the optimal value, worst value, mean value, and standard deviation of EGO, the FATA, GCRA, and TLGCRA remain consistent, all showing the optimal extreme solutions. With respect to quantitative metrics, detection efficacy, and extraction precision, the TLGCRA is superior to LPSO, SBKA, ESGJO, RLWOA, MSSCSO, PO, NRBO, AO, FATA, and HEOA. The individual with the best fitness performance selected from the population is the dominant male GCR, which is defined as the teacher, and the high-quality solution to the problem, corresponding to this individual, serves as the teaching goal for the entire population. This mechanism core realizes the knowledge transfer effect within the population. The remaining individuals in the population take the teacher’s high-quality solution as the guide, dynamically update their positions in the solution space, and gradually narrow the distance from the global optimal solution, thereby significantly reducing the probability of invalid exploration and improving search efficiency. Meanwhile, to avert homogenization during population evolution and preserve the diversity of the search procedure, this paradigm integrates a differentiated instructional strategy and stochastic perturbation factors. By simulating individual differences and independent exploration behaviors of different students in the learning process, it breaks the singularity of population convergence. For $f_{5}$, $f_{6}$, and $f_{7}$, the TLGCRA not only achieves a marginal enhancement in quantitative metrics, detection efficacy, and extraction precision but also substantially outperforms competing algorithms. The overall design idea is to integrate the randomness of biological behaviors with the directional guidance of the teaching mechanism, realize the complementary cooperation of the two characteristics, and thus effectively solve the core problems faced by traditional optimization algorithms, such as sluggish convergence rate, susceptibility to premature convergence, and suboptimal precision of the final solution.

For multimodal functions, their solution domain encompasses multiple local extrema, and the solution quality of certain local extrema approximates that of the global extremum. They feature multiple peaks and valleys, with these local extrema capable of forming multiple optimal solution traps. Owing to the interference of local extrema, conventional optimization algorithms are prone to becoming trapped at local extrema throughout the iteration procedure and fail to escape the traps to seek the global optimal solution. Hence, multimodal functions are frequently employed to evaluate the global exploration capacity and population diversity preservation capability of algorithms. Accordingly, the core demands for multimodal algorithms are to preserve population diversity, avert individual homogenization, break free from local extremum traps, realize an efficient search across the global domain, and precisely pinpoint the global optimal solution, rather than remaining at local suboptimal solutions. For $f_{8}$ and $f_{10}$, the optimal value, worst value, mean value, and standard deviation of the SBKA, ESGJO, RLWOA, MSSCSO, PO, NRBO, EGO, FATA, GCRA, and TLGCRA remain consistent, all showing the optimal extreme solutions and being superior to AO and HEOA. The TLGCRA combines the foraging, territorial competition and reproduction behaviors of the GCRA population with the teacher–student learning mechanism in an organic manner, thereby guiding advantageous individuals, adjusting the population orientation, preventing entrapment in local optima, and enhancing solution precision. For $f_{9}$, $f_{11}$ and $f_{12}$, the TLGCRA is superior to LPSO, SBKA, ESGJO, RLWOA, MSSCSO, PO, NRBO, EGO, AO, FATA, HEOA, and GCRA with respect to quantitative metrics, detection efficacy, and extraction precision. The TLGCRA exhibits robust dependability and utility in probing the solution domain, achieving comprehensive global search, strengthening collective cooperation and directional steering, discovering accurate optimal solutions, and avoiding early convergence. Taking advantage of the decentralized foraging characteristic of the GCRA population and combining the directional guidance mechanism of the teaching–learning mechanism, the TLGCRA is neither confined to blind search in local regions nor neglects potential high-performance solution domains. Regardless of the complexity of the problem domain, the proposed algorithm can systematically accomplish global scanning, establishing a foundation for subsequent optimization. At the same time, the territory competition and random foraging behaviors of the GCRA ensure the extensive traversal of the solution space by the population, and the student mutual learning link in the teaching mechanism further expands the exploration range through information interaction between individuals. The practicality of this design is that the algorithm can quickly cover most areas of the solution space in the early stage of iteration, avoiding falling into local optimal traps due to a narrow exploration range. The logic of the TLGCRA for achieving accurate optimization is as follows: In the early stage, it locks in potential optimal solution regions through global exploration. In the middle stage, it conducts refined mining of high-quality regions through the directional guidance of teachers and the local foraging mechanism of GCRA. In the later stage, it progressively converges toward the global optimal solution or high-quality approximate solution via fitness selection criteria. Regarding its solution precision, in comparison to the baseline GCRA and conventional optimization algorithms, the final solution yielded by the TLGCRA is more proximate to the theoretical global optimum, accompanied by a reduced error margin. Premature convergence stands as a common deficiency across optimization approaches. It manifests that the population drifts into local optima at the initial search stage and loses the capacity to evolve toward the global optimal solution thereafter. In spite of this, the essential working rule for the TLGCRA to elude premature convergence lies in the coordinated structure of exploration and exploitation. Directional orientation of the teaching mechanism hastens the exploitation of high-potential regions. Probabilistic interference elements from the GCRA, as well as discriminative learning among participants, consistently preserve population diversity and curb the emergence of individual homogenization. This favorable coordination allows the algorithm to keep its evolutionary potential in all iterative steps and avoid premature stagnation. It also demonstrates the algorithm’s steady performance.

The solution domain of fixed-dimensional multimodal functions exhibits the core attributes of both function types: it not only encompasses multiple local extrema of multimodal functions but also features the distinct global extremum of unimodal functions. This function type realizes efficient regulation of problem complexity through fixed-dimensional parameter design while eliminating the disruption of dimensional dynamic variations on algorithm performance assessment. The core evaluation demands for optimization algorithms lie in validating the algorithm’s capacity to mitigate performance deterioration caused by increasing dimensions alongside the algorithm’s global stability and resilience, the consistency of search outcomes across the solution domain, the ability to disentangle high-dimensional variables, the capacity to precisely orient search directions, adaptability to the dimensionality curse and the capability to strike a balance between global exploration and local exploitation. For $f_{13}$, $f_{14}$, $f_{15}$, $f_{16}$ $f_{17}$, $f_{21}$, $f_{22}$ and $f_{23}$, the best, worst, and mean fitness values of the TLGCRA remain consistent across all runs, yielding optimal extreme solutions and outperforming LPSO, SBKA, ESGJO, RLWOA, MSSCSO, PO, NRBO, EGO, AO, FATA, HEOA and GCRA with respect to quantitative performance metrics, search effectiveness, and extraction precision. The TLGCRA demonstrates robust stability and resilience, effectively guiding and adjusting the initial population distribution, consistently converging toward the optimal solution and reducing algorithmic variability. For $f_{18}$, $f_{19}$ and $f_{20}$, the TLGCRA exhibits pronounced superiority and operability with respect to quantitative metrics, detection efficacy and extraction precision. The TLGCRA leverages the directional guidance capability of the teaching–learning mechanism, which neither confines the search to blind exploration within local regions nor ignores potential high-quality solution domains. It persistently seeks the optimal solution to guarantee that it does not become trapped in local optima. The directional guidance of instructors enables the population to evade blind exploration and rapidly approach the optimal solution domain. Learners’ mutual learning integrated with the stochastic perturbation of the GCRA effectively sustains population diversity and averts entrapment in local optima. Meanwhile, the equilibrium between local exploitation and global exploration enhances the quality of the ultimate solution.

### 4.5. Convergence Analysis

**Figure 1:**
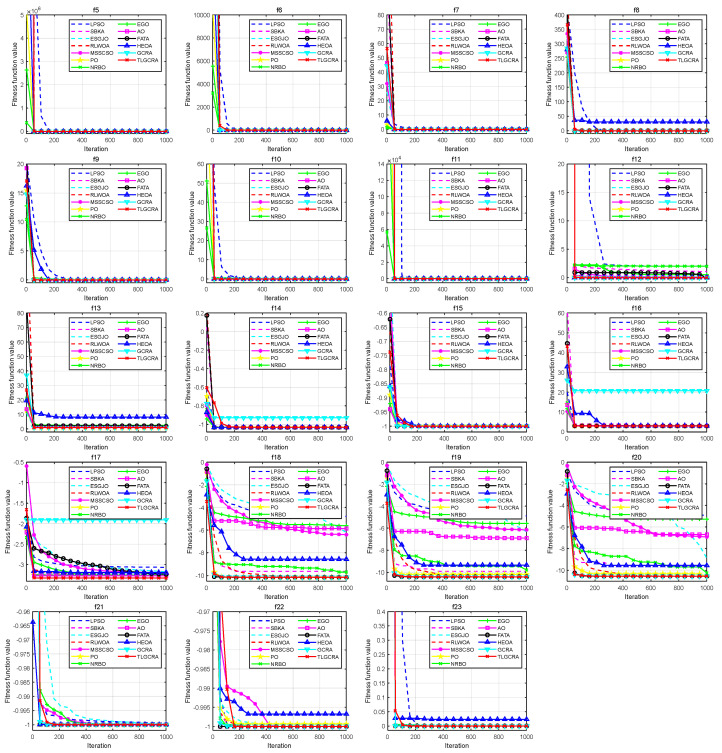
Convergence curves of TLGCRA and comparison algorithms for benchmark functions.

Figure 1 illustrates the comparative outcomes of the benchmark function gathering curves between the TLGCRA and various comparative algorithms. The optimization efficiency and computational accuracy of each algorithm can be comprehensively evaluated based on the steepness of curve decline, the duration of the iterative stable period, and the amplitude of numerical oscillation. Meanwhile, the optimization performance of these algorithms can be quantitatively analyzed by integrating three core indicators, convergence speed, optimal fitness value, and iteration number. Among them, the iteration count required for an algorithm to reach a steady state can directly characterize the convergence speed, the final optimal fitness value can intuitively reflect the algorithm’s solution accuracy, and the gap between the optimal value and the mean value of the algorithm can effectively determine the overall optimization performance. An overly broad disparity reveals that the approach is susceptible to premature convergence. Meanwhile a slight and gradually diminishing difference reflects that the algorithm reaches a favorable trade-off between global exploration and local exploitation capacities. One can clearly recognize from the convergence curves that the TLGCRA delivers remarkable merits in practical performance. At the initial iterative stage, the curve holds a steeper falling gradient and the objective value drops rapidly. This helps the algorithm draw near to the optimal search zone in a short time with a strengthened early global search ability and keeps away from local optimal traps effectively. Meanwhile, the algorithm requires fewer iteration steps to reach steady-state convergence, featuring lower iteration overhead and higher optimization efficiency. It can finally converge to a superior fitness value, with steady-state solution accuracy far outperforming 12 comparative algorithms, such as LPSO and MSSCSO, and it has no problems of stagnation in the later iteration stage or inability to achieve continuous optimization. In the tests of unimodal and multimodal benchmark functions, the TLGCRA performs excellently in all performance indicators, with comprehensive optimization performance significantly superior to other comparative algorithms. Such outstanding performance stems from the integration of innovative mechanisms of the algorithm. On the basis of the regional foraging, territory competition and elite guidance mechanisms of the traditional GCRA, the TLGCRA incorporates the learning guidance mechanism of the teaching–learning-based optimization paradigm to improve local exploitation performance. It discards the GCRA’s position update rule that solely depends on random step sizes, and develops a directional update strategy that integrates the position of teacher solutions and the average fitness position of the population, balancing both accelerated convergence speed and global search efficacy. In tandem with this, the formulated algorithm implements a bidirectional peer learning framework to facilitate inter-individual information exchange via stochastic pairing of agents, and enhances the search mechanism through random perturbation strategies, which successfully alleviates the inherent flaws of the original GCRA implementation, such as population homogenization during late-stage iterations and vulnerability to local stagnation. This differentiated learning paradigm not only guides the population to converge toward the global optimum but also consistently preserves population diversity, granting the proposed method the ability to avoid local entrapment even in the latter phases of the optimization run.

In summary, the TLGCRA is not a simple superposition of multiple mechanisms. It addresses the limitations of the baseline algorithm’s random search mechanism through the directional guidance of teaching and learning, effectively balances the core contradiction between fast convergence and global exploration, and ultimately achieves all-round improvements in the algorithm’s rate of convergence, solution accuracy and resistance to premature convergence.

### 4.6. Boxplot Analysis

**Figure 2:**
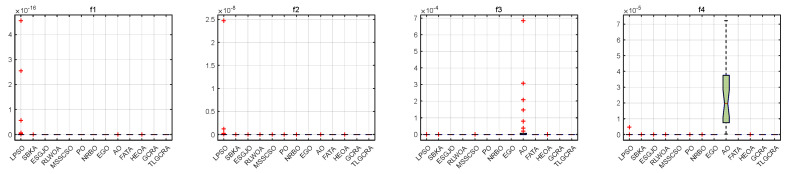

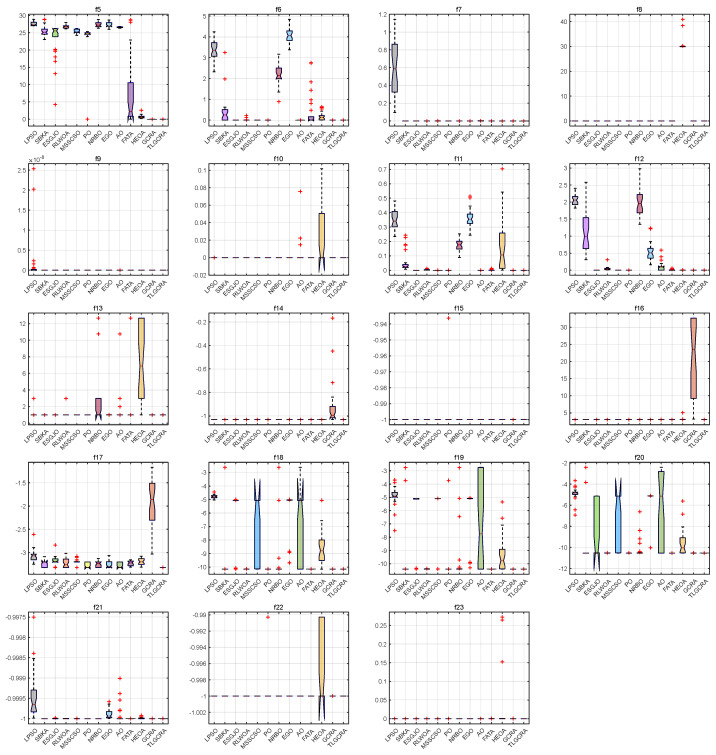
Boxplots of TLGCRA and comparison algorithms processing benchmark functions.

Figure 2 presents boxplot comparisons of the TLGCRA against baseline algorithms on benchmark test functions. Boxplots evaluate the consistency and robustness of solutions generated by each algorithm, uncover evolutionary trends in solution-set diversity, and describe the variability in candidate solutions in the population. The standard deviation serves as an intuitive indicator of an algorithm’s sensitivity to initial population distributions and parameter disturbances. A monotonic decline in standard deviation denotes sound convergence behavior and drives the entire population toward the global optimum. Abrupt variations or fluctuations in standard deviation imply potential stagnation in local optima or the necessity of parameter adjustment. The stability and robustness of each approach can be evaluated via joint analysis of standard deviation and worst-case fitness values. Low standard deviation, together with worst-case values close to the global optimum, signifies strong adaptability to stochastic factors and initial settings. Standard deviation also serves as a reliable metric for assessing the uniformity of solution distribution and validating the effects of improved strategies on solution-set diversity, with high extensibility. Experimental results illustrate that on unimodal benchmark functions, the TLGCRA achieves superior standard deviation and dispersion indices relative to competing algorithms. The proposed method effectively alleviates issues, including parameter sensitivity, insufficient optimization precision, susceptibility to overfitting, and weak dynamic adaptability, thus presenting remarkable practical applicability. On multimodal functions, the TLGCRA also exhibits distinct advantages in standard deviation and dispersion metrics. By virtue of the dispersed foraging traits inherited from the GCRA and the directional guidance embedded in the teaching–learning mechanism, the TLGCRA avoids confined local-region searches and neglect of promising high-quality solution regions. Irrespective of solution space complexity, the TLGCRA performs systematic global scanning and establishes a reliable foundation for subsequent optimization procedures. The core design philosophy of the TLGCRA lies in the integration of stochastic biological behavioral characteristics with directional guidance from the teaching–learning mechanism. Such integration generates complementary synergies between the two mechanisms and successfully addresses critical drawbacks of conventional optimization algorithms, such as sluggish convergence speeds vulnerability to premature convergence and suboptimal precision of final solutions. In summary, the TLGCRA presents remarkable superiority and adaptability in achieving favorable standard deviation, dispersion, stability, and robustness. Comprehensive advantages over conventional algorithms verify that the TLGCRA ensures full global coverage of the solution space and precisely locates optimal solutions through efficient synergistic search when addressing complex optimization problems. This framework effectively circumvents premature convergence and possesses tangible practical application value. Additionally, the TLGCRA exhibits exceptional practical value and consistent performance in harmonizing global exploration with local exploitation, broadening solution-domain coverage, precisely pinpointing potential optimal domains, reinforcing local exploitation, and effectively enhancing solution precision.

### 4.7. Wilcoxon Rank-Sum Test

The Wilcoxon rank-sum test is a nonparametric statistical approach designed for matched datasets. It allows for quantifying the magnitude of the overall performance difference between the TLGCRA and other competing algorithms, eliminating the requirement to depend on pre-existing assumptions about data distribution [34]. A specific criterion p<0.05 denotes a statistically significant difference, whereas a higher *p*-value indicates a non-significant difference, and the notation N/A is used to represent the not applicable scenario. Table 4 displays the comparative outcomes of the Wilcoxon rank-sum test with respect to *p*-values across the collection of benchmark functions. The findings reveal that the TLGCRA exhibits robust stability and dependability in producing genuine and reliable outcomes rather than producing infrequent false optimal values by accident.

## 5. TLGCRA for Tackling Engineering Designs

To verify the adaptability and practical applicability of the Teaching and Learning Mechanism integrated with the greater cane rat algorithm, the algorithm was employed to address constrained practical engineering design tasks prevalent in industrial settings. These problems include the three-bar truss [35], speed reducer [36], gear train [37], pressure vessel [38], multi-disk clutch brake [39], and tubular column [40]. The six engineering optimization problems involve complex constraint conditions, which impose strict requirements on the engineering feasibility of solution schemes and necessitate rigorous feasibility verification of optimization results. The six constrained engineering optimization cases adopted in this paper are well-recognized classical benchmark models in the research field. The design variables, constraint equations and parameter values of all cases are referenced from the mainstream classical literature on constrained optimization, with standard constraint intervals and variable value ranges universally adopted in the industry. Meanwhile, the source of constraint conditions and the boundary ranges of basic parameters for each case are clearly defined in the manuscript, which ensures the standardization and uniformity of experimental models and provides a reliable and reproducible research foundation for the comparative experimental results of the proposed algorithm.

### 5.1. Three-Bar Truss Design

**Figure 3:**
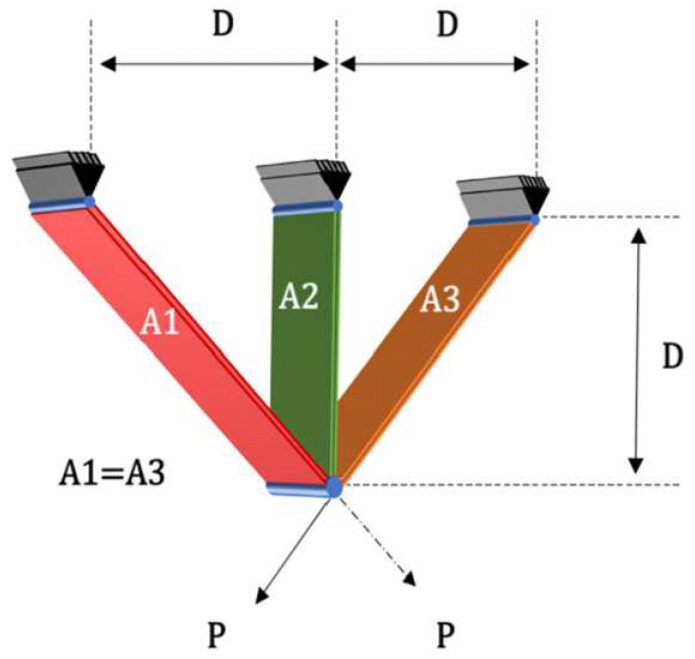
Sketch map of the three-bar truss design.

The central optimization objective for this engineering design task is aimed at minimizing the overall mass of the structural system depicted in Figure 3. A pair of cross-sectional parameters is selected as decision variables, and the associated mathematical model is established below:

Consider

(18) $$x=[x_{1}\;x_{2}]=[A_{1}\;A_{2}]$$

Minimize

(19) $$f(x)=(2\sqrt{2}x_{1}+x_{2})\times l$$

This is subject to

(20) $$g_{1}(x)=\frac{\sqrt{2}x_{1}+x_{2}}{\sqrt{2}x_{1}^{2}+2x_{1}x_{2}}P-\sigma \leq 0$$

(21) $$g_{2}(x)=\frac{x_{2}}{\sqrt{2}x_{2}+2x_{1}x_{2}}P-\sigma \leq 0$$

(22) $$g_{3}(x)=\frac{1}{\sqrt{2}x_{2}+x_{1}}P-\sigma \leq 0$$

(23) $$l=100\;\mathrm{cm},\;P=2\;\mathrm{kN}/{\mathrm{cm}}^{2},\;\sigma =2\;\mathrm{kN}/{\mathrm{cm}}^{2}$$

The variable range is

(24) $$0\leq x_{1},x_{2}\leq 1$$

Table 5 illustrates comparative outcomes of various algorithms for the three-bar truss engineering task. The cross-sectional dimensions of a pair of truss members, labeled as $A_{1}$ and $A_{2}$, act as decision variables with the goal of reducing the overall structural mass while meeting mechanical restrictions encompassing stress and displacement thresholds. Unlike conventional algorithms relying on fixed step sizes, the TLGCRA adopts an adaptive step-size adjustment strategy; a larger step size is employed during the global exploration phase to traverse broader solution spaces, while a smaller step size is used in the local refinement phase to enhance optimization precision. By integrating the teaching–learning-guided directional optimization with the foraging reproduction collaborative mechanism of giant cane rats, the TLGCRA attains an equilibrium between global search and local utilization, successfully preventing entrapment in local optima. Constraint handling adopts a feasible region mapping approach without complex penalty functions, ensuring all solutions remain within feasible bounds, thus significantly improving optimization efficiency and solution quality. The global optimal solution obtained by the TLGCRA corresponds to the decision variable values of 0.78699 and 0.41384, with the optimized total weight of the three-bar truss calculated as 263.8786.

### 5.2. Tubular Column

**Figure 4:**
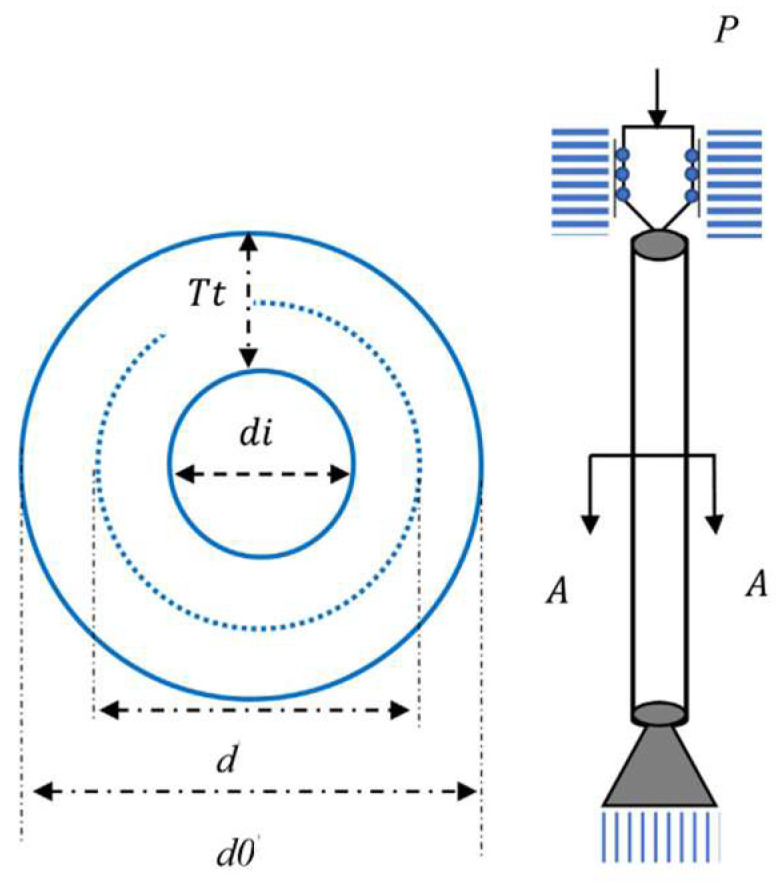
Sketch map of the tubular column design.

The main objective of this design problem is to minimize the material and construction costs, as shown in Figure 4. There are two decision variables: the mean diameter of the column (d) and the thickness of tube (t). The mathematical model is described as follows.

Consider

(25) $$x=[x_{1}\;x_{2}]=[d\;t]$$

Minimize

(26) $$f(x)=9.82x_{1}x_{2}+2x_{1}$$

This is subject to

(27) $$g_{1}(x)=\frac{P}{\pi x_{1}x_{2}\sigma_{y}}-1\leq 0$$

(28) $$g_{2}(x)=\frac{8PL^{2}}{\pi^{3}Ex_{1}x_{2}(x_{1}^{2}+x_{2}^{2})}-1\leq 0$$

(29) $$g_{3}(x)=\frac{2.0}{x_{1}}-1\leq 0$$

(30) $$g_{4}(x)=\frac{x_{1}}{14}-1\leq 0$$

(31) $$g_{5}(x)=\frac{0.2}{x_{2}-1}\leq 0$$

(32) $$g_{6}(x)=\frac{x_{2}}{0.8}-1\leq 0$$

(33) $$\sigma_{y}=500\;\mathrm{kgf}/{\mathrm{cm}}^{2},\;E=0.85\times 10^{6}\;\mathrm{kgf}/{\mathrm{cm}}^{2},\;P=2500\;\mathrm{kgf},\;L=250\;\mathrm{cm}$$

The variable range is

(34) $$2\leq x_{1}\leq 14,\;0.2\leq x_{2}\leq 0.8$$

Table 6 displays comparative outcomes for the tubular column engineering design task. The TLGCRA employs a two-stage optimization strategy integrated with the teaching–learning mechanism, preventing unidirectional population convergence and local optima entrapment, thereby facilitating efficient resolution of constrained single-objective optimization for tubular column design. The problem takes the column’s average diameter (d) and wall thickness (t) as decision variables, with the core objective of minimizing material and construction costs. The TLGCRA ensures rapid convergence to the global optimum while satisfying compressive strength and Euler buckling stability constraints. In the teaching phase, the global optimal position guides population evolution to enhance convergence efficiency; in the learning feedback phase, adaptive weights balance population diversity and local exploitation. Out-of-bounds or constraint-violating individuals undergo random reinitialization or opposition-based correction, ensuring all solutions remain feasible. This design balances global exploration and local refinement, achieving superior optimization performance. The global optimal solution obtained by the TLGCRA is quantitatively d characterized by the decision variable values of *d* = 5.4637 and *t* = 0.29133, corresponding to a minimum cost of 26.5263.

### 5.3. Speed Reducer

**Figure 5:**
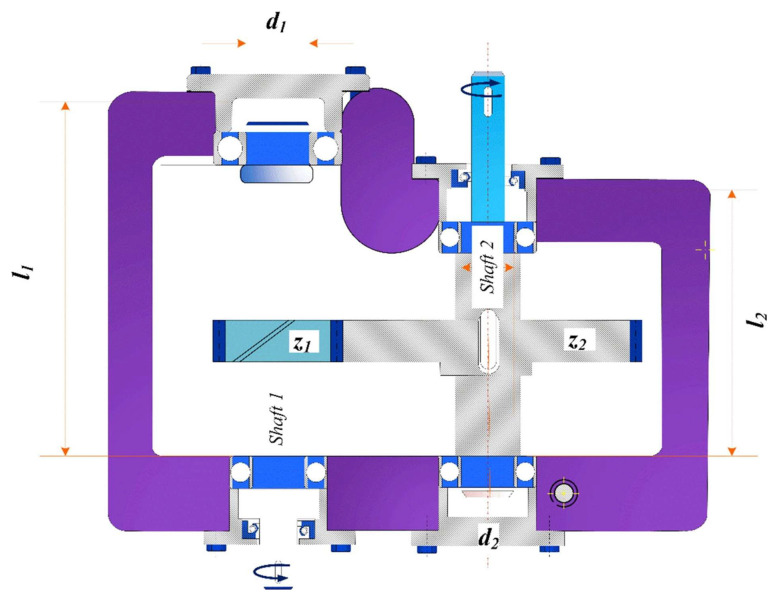
Sketch map of the speed reducer design.

The central optimization objective of this engineering design problem is to reduce the total weight of the gearbox, as depicted in Figure 5. There exist seven design variables, including tooth face width (b), gear module (m), pinion tooth count (z), distance between the bearings on the first shaft ($l_{1}$), distance between the bearings on the second shaft ($l_{2}$), diameter of the first shaft ($d_{1}$) and diameter of the second shaft ($d_{2}$). The corresponding mathematical model is provided below.

Consider

(35) $$x=[x_{1}\;x_{2}\;x_{3}\;x_{4}\;x_{5}\;x_{6}\;x_{7}]=[b\;m\;z\;l_{1}\;l_{2}\;d_{1}\;d_{2}]$$

Minimize

(36) $$\begin{array}{l} f(x)=0.7854x_{1}x_{2}^{2}(3.3333x_{3}^{2}+14.9334x_{3}-43.0934)\\ \;\;-1.508x_{1}(x_{6}^{2}+x_{7}^{2})+7.4777(x_{6}^{3}+x_{7}^{3})+0.7854(x_{4}x_{6}^{2}+x_{5}x_{7}^{2}) \end{array}$$

This is subject to

(37) $$g_{1}(x)=\frac{27}{x_{1}x_{2}^{2}x_{3}^{2}}-1\leq 0$$

(38) $$g_{2}(x)=\frac{397.5}{x_{1}x_{2}^{2}x_{3}}-1\leq 0$$

(39) $$g_{3}(x)=\frac{1.93x_{4}^{3}}{x_{2}x_{6}^{4}x_{3}}-1\leq 0$$

(40) $$g_{4}(x)=\frac{1.93x_{5}^{3}}{x_{2}x_{7}^{5}x_{3}}-1\leq 0$$

(41) $$g_{5}(x)=\frac{[(745x_{4}/x_{2}x_{3}{)}^{2}+16.9\times 10^{6}{]}^{1/2}}{110x_{6}^{3}}-1\leq 0$$

(42) $$g_{6}(x)=\frac{[(745x_{5}/x_{2}x_{3}{)}^{2}+157.5\times 10^{6}{]}^{1/2}}{85x_{7}^{3}}-1\leq 0$$

(43) $$g_{7}\left(x\right)=\frac{x_{2}x_{3}}{40}-1\leq 0$$

(44) $$g_{8}\left(x\right)=\frac{5x_{2}}{x_{1}}-1\leq 0$$

(45) $$g_{9}\left(x\right)=\frac{x_{1}}{12x_{2}}-1\leq 0$$

(46) $$g_{10}\left(x\right)=\frac{1.5x_{6}+1.9}{x_{4}}-1\leq 0$$

(47) $$g_{11}\left(x\right)=\frac{1.1x_{7}+1.7}{x_{5}}-1\leq 0$$

The variable range is

(48) $$2.6\leq x_{1}\leq 3.6,0.7\leq x_{2}\leq 0.8,17\leq x_{3}\leq 28,7.3\leq x_{4},x_{5}\leq 8.3,2.9\leq x_{6}\leq 3.9,5.0\leq x_{7}\leq 5.5$$

The comparative outcomes for the reducer engineering design task are displayed in Table 7. The optimization goal is defined to minimize the overall mass of the reducer, with seven key structural parameters defined as decision variables. The design procedure must comply with stringent constraints, including gear strength, transmission efficiency and structural space boundaries. Directional guidance derived from the teaching mechanism in the TLGCRA suppresses blind searching in the original GCRA and excludes unreasonable schemes with overlarge moduli without redundant iterations, thus accelerating convergence toward the global optimum. Feasible individuals with minimal weight act as formal teachers, and the candidate with the lowest constraint violation serves as a temporary teacher when no feasible solution exists. Learner individuals update seven-dimensional position vectors via teaching equations and random parameter swapping to maintain population diversity. A dynamic step-size framework integrates global exploration and local refinement, and adaptive constraint processing ensures the engineering manufacturability of final solutions, supporting direct utilization in industrial production. The global optimal solution achieved by the TLGCRA has the following quantitative indicators for the seven decision variables, 3.5213, 0.7001, 17, 7.3, 7.8013, 3.3531, and 5.2863, with the corresponding optimal total weight being 2995.4217.

### 5.4. Pressure Vessel

**Figure 6:**
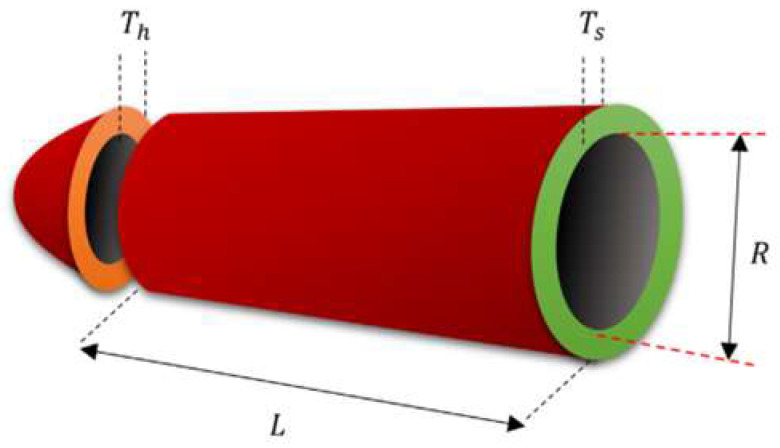
Sketch map of the pressure vessel design.

The central optimization objective of this engineering design task is to minimize the total fabrication cost, as depicted in Figure 6. This task encompasses four decision variables, namely the vessel shell thickness ($T_{s}$), inner radius (R), head thickness ($T_{h}$) and the length of the cylindrical section excluding the end head (L). The associated mathematical formulation is presented as follows:

Consider

(49) $$x=[x_{1}\;x_{2}\;x_{3}\;x_{4}\;]=[T_{s}\;T_{h}\;R\;L]$$

Minimize

(50) $$f(x)=0.6224x_{1}x_{3}x_{4}+1.7781x_{2}x_{3}^{2}+3.1661x_{1}^{2}x_{4}+19.84x_{1}^{2}x_{3}$$

This is subject to

(51) $$g_{1}(x)=-x_{1}+0.0193x_{3}\leq 0$$

(52) $$g_{2}(x)=-x_{2}+0.00954x_{3}\leq 0$$

(53) $$g_{3}(x)=-\pi x_{3}^{2}x_{4}-\frac{4}{3}\pi x_{3}^{3}+1296000\leq 0$$

(54) $$g_{4}(x)=x_{4}-240\leq 0$$

The variable range is

(55) $$0\leq x_{1},x_{2}\leq 99,\;10\leq x_{3},x_{4}\leq 200$$

The comparative outcomes for the pressure vessel engineering design task are displayed in Table 8. The four key design parameters of the pressure vessel are utilized as the positional parameters of the cane rats within the TLGCRA population, and the fabrication cost expression is designated as the optimization objective of the algorithm. An initial population is created stochastically, and the constraint-violating solutions are removed. The solution with the minimum cost in the current population is selected as the teacher, and the positions of other solutions are updated according to the teaching mechanism formula, thus guiding the candidate design schemes to move toward the direction of lower cost and constraint satisfaction. By means of information exchange among student solutions and combined with the scattered foraging characteristics of cane rats, the algorithm performs a small-step refined search in high-quality solution regions and large-step exploration in unexplored regions. In the global optimal solution derived from the TLGCRA, the quantitative values of the four decision variables are as follows, 0.7463, 0.3631, 41.3014 and 200, with the corresponding optimal cost being 5884.2153.

### 5.5. Gear Train Design

**Figure 7:**
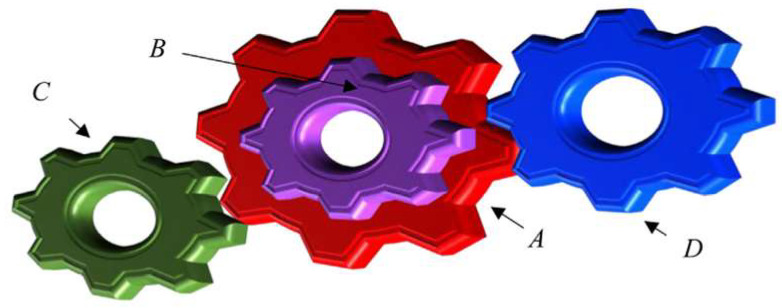
Sketch map of the gear train design.

The central optimization objective of this engineering design task is to identify the optimal gear tooth numbers and reduce the gear ratio expenditure, as depicted in Figure 7. This task encompasses four decision variables, specifically the gear tooth numbers of gears $n_{A}$, $n_{B}$, $n_{C}$ and $n_{D}$. The associated mathematical formulation is presented as follows:

Consider

(56) $$x=[x_{1}\;x_{2}\;x_{3}\;x_{4}]=[n_{A}\;n_{B}\;n_{C}\;n_{D}]$$

Minimize

(57) $$f(x)={\left(\frac{1}{6.931}-\frac{x_{3}x_{2}}{x_{1}x_{4}}\right)}^{2}$$

The variable range is

(58) $$12\leq x_{i}\leq 60,\;i=1,2,\ldots ,4$$

Table 9 displays the comparative outcomes of the gear train engineering design tasks. The tooth counts of the four gears are utilized as the positional parameters of the cane rat population within the algorithm, and the gear transmission ratio cost expression is employed as the optimization objective of the algorithm. The transmission ratio cost is typically associated with the complexity of machining the tooth counts, the utilization of gear materials, and the degradation of transmission efficiency. A smaller function value indicates a lower cost. At the same time, engineering constraints such as meshing, strength, and undercutting are converted into the constraint conditions of the algorithm, and the tooth number combinations that violate the constraints are penalized so that they are eliminated in the iteration. The TLGCRA combines the population’s scattered foraging characteristics of the cane rat algorithm and the directional guidance characteristics of the teaching optimization algorithm to complete the search for the optimal number of teeth in stages, avoiding traditional algorithms from falling into local optima. Among them, the hybrid mechanism of the TLGCRA can exactly solve the problem of strong variable coupling in gear tooth number optimization. In the global optimal solution obtained by the TLGCRA, the quantitative values of the four decision variables are as follows, 52, 30, 15 and 59, with the corresponding optimal cost being 4.5921 × 10^−14^.

### 5.6. Multiple-Disk Clutch Brake

**Figure 8:**
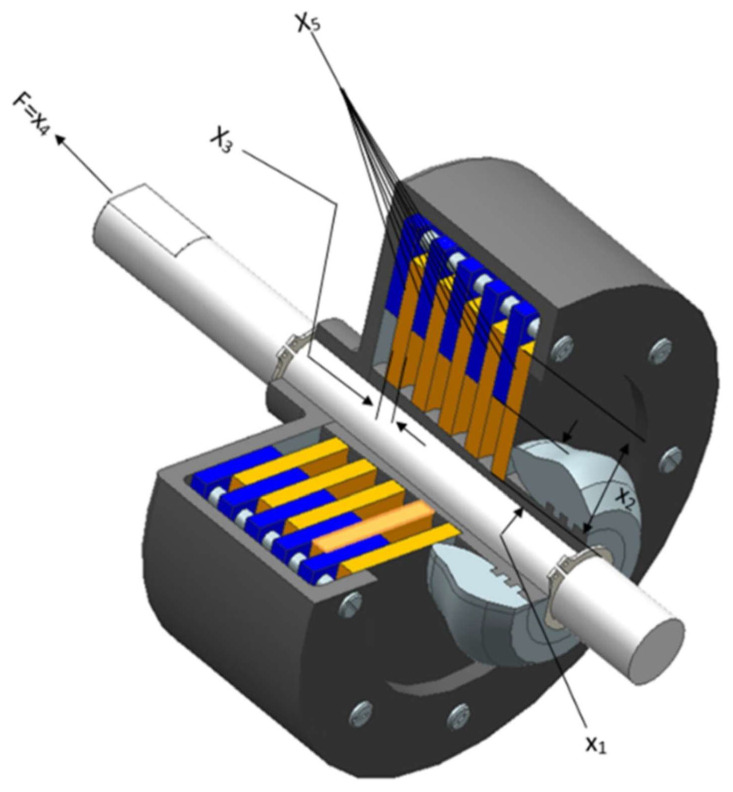
Sketch map of the multiple-disk clutch brake design.

The primary goal of this design task is to minimize the total mass of a multi-disk clutch brake design, as shown in Figure 8. This engineering problem involves five design variables, namely the disk thickness (t), internal radius ($r_{i}$), external radius ($r_{o}$), actuation force (F) and the count of friction interfaces (Z). The corresponding mathematical model is provided below.

Consider

(59) $$x=[x_{1}\;x_{2}\;x_{3}\;x_{4}\;x_{5}]=[r_{i}\;r_{0}\;t\;F\;Z]$$

Minimize

(60) $$f(x)=\pi t\rho (r_{0}^{2}-r_{i}^{2})(Z+1)$$

This is subject to

(61) $$g_{1}(x)=r_{0}-r_{i}-\Delta r\geq 0$$

(62) $$g_{2}(x)=l_{\max}-(Z+1)(t+\delta )\geq 0$$

(63) $$g_{3}(x)=p_{\max}+p_{rz}\geq 0$$

(64) $$g_{4}(x)=p_{\max}v_{sr\;\max}-p_{rz}v_{sr}\geq 0$$

(65) $$g_{5}(x)=v_{sr\;\max}-v_{sr}\geq 0$$

(66) $$g_{6}(x)=T_{\max}-T\geq 0$$

(67) $$g_{7}(x)=M_{h}-sM_{s}\geq 0$$

(68) $$g_{8}(x)=T\geq 0$$

(69) $$M_{h}=\frac{2}{3}\mu FZ\frac{r_{0}^{3}-r_{i}^{3}}{r_{0}^{2}-r_{i}^{2}}$$

(70) $$p_{rz}=\frac{F}{\pi (r_{0}^{2}-r_{i}^{2})}$$

(71) $$v_{sr}=\frac{2\pi n(r_{0}^{3}-r_{i}^{3})}{90(r_{0}^{2}-r_{i}^{2})}$$

(72) $$T=\frac{I_{z}\pi n}{30(M_{h}+M_{f})}$$

(73) $$\Delta r=20\;\mathrm{mm},\;I_{z}=55\;{\mathrm{kgmm}}^{2},\;p_{\max}=1\;\mathrm{Mpa},\;F_{\max}=1000\;N$$

(74) $$T_{\max}=15\;s,\;\mu =0.5,\;s=1.5,\;M_{s}=40\;\mathrm{Nm}$$

(75) $$M_{f}=3\;\mathrm{Nm},\;n=250\;\mathrm{rpm}$$

(76) $$v_{sr\;\max}=10\;m/s,\;l_{\max}=30\;\mathrm{mm},\;r_{i\;\min}=60$$

(77) $$r_{i\;\max}=80,\;r_{o\;\min}=90$$

(78) $$r_{o\;\max}=110,\;t_{\min}=1.5,\;t_{\max}=3,\;F_{\min}=600$$

(79) $$F_{\max}=1000,\;Z_{\min}=2,\;Z_{\max}=9$$

Table 10 displays comparative outcomes for the multi-disk clutch brake engineering design task. The optimization goal is to reduce the overall mass, with five decision parameters: disk thickness, internal radius, external radius, actuation force, and count of friction interfaces. Rigorous engineering constraints cover torque transmission capability, friction wear limitations, temperature rise constraints, and structural space boundaries. The TLGCRA converts five design variables into optimization parameters and conducts hybrid iterations of foraging exploration and a teaching-guided search. This strategy identifies the minimum-weight design while satisfying all constraints. The initial population is stochastically created within permissible engineering intervals. The feasible solution with the minimal objective value serves as the teacher individual, and other individuals update parameters through the teaching mechanism. The TLGCRA adopts adaptive step-size adjustment: large steps enhance global exploration in dispersed populations, and small steps strengthen local refinement in high-quality solution regions. Random information exchange among individuals prevents trapping in local optima. Iterations terminate at the preset maximum generation or objective function stagnation. The directional teaching guidance and adaptive step-size mechanism achieve a balanced trade-off between global exploration and local exploitation, and constraint handling guarantees that the final design satisfies engineering manufacturing specifications. In the global optimal solution derived from the TLGCRA, the quantitative values of the five decision variables are as follows, 70, 90, 1, 958 and 2, with the corresponding optimal weight being 0.2351.

## 6. Conclusions and Future Works

The TLGCRA draws on the innate foraging and reproductive behavioral mechanisms of the giant cane rat population, innovatively introducing a teaching mechanism to optimize the population update strategy, which effectively balances the algorithm’s global exploration ability and local exploitation performance. This paper verifies the algorithm performance through systematic experiments, prospects future research directions combined with application scenarios, and provides a reference for the further optimization and popularization application of the algorithm. In the research, 23 standard benchmark test functions are selected to compare the TLGCRA with mainstream intelligent optimization algorithms and the traditional GCRA, and performance evaluation is carried out from three dimensions: convergence precision, convergence rate and stability. The experimental findings demonstrate that the introduced teaching mechanism can accelerate the population convergence speed and effectively solve the problem that traditional algorithms tend to get trapped in local optimal stagnation. In multimodal function test scenarios, the algorithm can preserve favorable population diversity and possess superior global exploration performance in high-dimensional complex solution spaces. The practical utility of the TLGCRA is verified through real-world engineering design tasks such as mechanical structure optimization and path planning, and the algorithm exhibits reliable and efficient optimization performance in complex practical scenarios. Statistical test results further confirm that notable performance gaps exist between the TLGCRA and the comparative algorithms, verifying the rationality and superiority of the design integrating the teaching mechanism. This paper proposes future research ideas from three directions, algorithm improvement, application expansion and cross-domain integration. At the algorithm optimization level, the dynamic adaptive capability of the teaching mechanism can be strengthened, a multi-strategy integration framework can be constructed, and a dimensional decomposition co-evolution strategy can be designed for high-dimensional problems. At the application expansion level, the TLGCRA can be extended to more complex engineering fields through custom parameters and constraint conditions. At the cross-domain integration level, the algorithm can be combined with deep learning to expand into the fields of multi-objective optimization and dynamic optimization, and algorithm deployment can be realized relying on parallel hardware architectures to adapt to the demands of real-time optimization scenarios.

In summary, the teaching–learning-based greater cane rat algorithm (TLGCRA) achieves performance breakthroughs through mechanism innovation, showing remarkable advantages in both theoretical tests and practical applications. Further algorithm optimization, scenario expansion and cross-domain integration in subsequent research are expected to improve its optimization efficiency and application coverage, providing more efficient intelligent algorithm solutions for various complex optimization problems. However, the optimization performance of the TLGCRA is only verified by traditional classical benchmark functions and multi-constrained engineering examples. Although its convergence superiority and engineering adaptability can be initially reflected, the coverage of the adopted test functions is limited, and the comprehensive performance of the algorithm still needs further verification in a more authoritative and complex standard test system. Following the mainstream evaluation paradigm in the field of metaheuristic optimization, future research will conduct supplementary numerical experiments based on the CEC2017 benchmark function set. Relying on the high-dimensional, multimodal and complex ill-conditioned function clusters contained in CEC2017, the global search stability, high-dimensional robustness and generalization ability of the TLGCRA will be further explored. Meanwhile, future research will broaden the dimension of comparative experiments, introduce the Improved Carnivorous Plant Algorithm (I-CPA) [90] and the Multi-Strategy Tunicate Swarm Algorithm (MS-TSA) [91] as new comparative baselines, and expand the comparison scope with current mainstream advanced optimization algorithms. A more comprehensive and multi-level algorithm performance evaluation system will be established to verify the superiority, effectiveness and generalization performance of the proposed TLGCRA with sufficient and convincing experimental results.

## Figures and Tables

**Table 1:** The fundamental difference between the TLGCRA and other hybrid approaches.

Comparative Dimension	PSO	DE	CMA-ES	GCRA	TLGCRA
Core search mechanism	Particles dynamically adjust their velocities and positions based on individual historical optimal and global optimal positions, and search for the optimal solution iteratively through group collaboration	Differential mutational scheme guided by discrepancies among population members and the selective filtering process	Covariance matrix adaptation, Gaussian sampling, guided by distribution parameters update	Simulate biological habits of greater cane rats, two-stage bionic evolution, with weak internal information interaction	Integrate TLBO’s teaching–learning mechanism into GCRA’s two-stage framework; realize adaptive global exploration guided by elites and efficient local exploitation via peer learning, with clear and complementary mechanism design
Parameter sensitivity	Increased inertia weight and acceleration coefficient settings significantly alter the convergence performance of the optimization process	Scaling factor and crossover probability directly affect the solution accuracy	Low, but with complex internal parameter learning	Medium, fixed seasonal factor and behavioral parameters, limited adjustment flexibility	Ultra-low, all key parameters are adaptive, self-adjust with iteration progress, no manual tuning required
Determinacy of solution	Pronounced local stochasticity and unregulated velocity perturbation	Substantial local stochastic behavior and randomized creation of differential vectors	Regulated stochasticity and deterministic distribution adjustment driven by elite individuals	Strong randomness from simulated behaviors; lack of directional guidance, unstable convergence	Controlled randomness with clear guidance; TLBO elite learning provides deterministic direction, ensuring stable and predictable convergence
Utilization mode of the solution space	Individual particle tracing tactic inclined to being enclosed in local extremal areas	Leveraging differential vectors and deficient sampling coverage across high-dimensional solution spaces	Adaptive distribution sampling, good high-dimensional coverage but high computational cost	Two-stage exploration–exploitation, but weak local exploitation, prone to insufficient development of high-quality regions	Integrated exploration–exploitation mechanism, full coverage of solution space, strong local exploitation capability, excellent performance in high-dimensional and multimodal spaces
Robustness to noise	At moderate levels particle velocity is vulnerable to noise perturbation	Medium, differential variation is sensitive to noise	Strong, but with high computational overhead	Medium, individual behavioral updates are easily affected by noise, lack of stable guidance	Excellent, TLBO elite guidance suppresses noise impact, adaptive parameters enhance stability, strong anti-interference ability
Adaptation scenario	Dimensional ranges from low to intermediate, faint stochastic perturbation, fully continuous search problem	Low and intermediate dimensionality continuously differentiable and well-behaved disturbance-free optimization task	High-dimensional, non-smooth, noisy problem, but with high computational cost	Moderate-dimensional slightly noisy smooth problems with limited adaptability to complex scenarios	Versatile for all scenarios, including high-dimensional, non-smooth, noisy, constrained, and multimodal engineering problems, with excellent adaptability

**Table 2:** Benchmark functions.

Benchmark Test Functions	Dim	Range	$f_{\min}$
$f_{1}=\sum_{i=1}^{n}x_{i}^{2}$	30	[−100,100]	0
$f_{2}(x)=\sum_{i=1}^{n}\left|x_{i}\right|+\prod_{i=1}^{n}\left|x_{i}\right|$	30	[−10,10]	0
$f_{3}(x)=\sum_{i=1}^{n}{\left(\sum_{j=1}^{i}x_{j}\right)}^{2}$	30	[−100,100]	0
$f_{4}(x)=\max_{i}\{|x_{i}|,1\leq i\leq n\}$	30	[−100,100]	0
$f_{5}(x)=\sum_{i=1}^{n-1}\left[100{\left(x_{i+1}-x_{i}^{2}\right)}^{2}+{\left(x_{i}-1\right)}^{2}\right]$	30	[−30,30]	0
$f_{6}(x)=\sum_{i=1}^{n}{\left([x_{i}+0.5]\right)}^{2}$	30	[−100,100]	0
$f_{7}(x)=\sum_{i=1}^{n}ix_{i}^{4}+random[0,1)$	30	[−1.28,1.28]	0
$f_{8}(x)=\sum_{i=1}^{n}\left[x_{i}^{2}-10\cos(2\pi x_{i})+10\right]$	30	[−5.12,5.12]	0
$\begin{array}{l} f_{9}(x)=-20\exp\left(-0.2\sqrt{\frac{1}{n}\sum_{i=1}^{n}x_{i}^{2}}-\exp\left(\frac{1}{n}\sum_{i=1}^{n}\cos2\pi x_{i}\right)\right)\\ +20+e \end{array}$	30	[−32,32]	0
$f_{10}(x)=\frac{1}{4000}\sum_{i=1}^{n}\left(x_{i}^{2}\right)-\prod_{i=1}^{n}\cos\left(\frac{x_{i}}{\sqrt{i}}\right)+1$	30	[−600,600]	0
$\begin{array}{l} f_{11}(x)=\frac{\pi }{n}\left\{10\sin^{2}(\pi y_{1})+\sum_{i=1}^{n-1}\begin{array}{l} {\left(y-1\right)}^{2}[1+10\sin^{2}(\pi y_{1})]\\ +{\left(y_{n}-1\right)}^{2} \end{array}\right\}\\ +\sum_{i=1}^{n}u(x_{i},10,100,4)\\ y_{i}=1+\frac{x_{i}+1}{4}\\ u(x_{i},a,k,m)=\left\{\begin{array}{l} k{\left(x_{i}-a\right)}^{m},x^{i}>a\\ 0,-a\leq x_{i}\leq a\\ k{\left(-x_{i}-z\right)}^{m},x_{i}<a \end{array}\right. \end{array}$	30	[−50,50]	0
$\begin{array}{l} f_{12}(x)=0.1\left\{\sin^{2}\left(3\pi x_{1}+\sum_{i=1}^{n}\begin{array}{l} {\left(x_{i}-1\right)}^{2}[1+\sin^{2}(3\pi x_{i}+1)]\\ +{\left(x_{n}-1\right)}^{2}[1+\sin^{2}(2\pi x_{n})] \end{array}\right)\right\}\\ +\sum_{i=1}^{n}u(x_{i},5,100,4) \end{array}$	30	[−50,50]	0
$f_{13}(x)={\left(\frac{1}{500}+\sum_{j=1}^{25}\frac{1}{j+\sum_{i=1}^{2}{\left(x_{i}-a_{ij}\right)}^{6}}\right)}^{-1}$	2	[−65,65]	0.998
$f_{14}(x)=4x_{1}^{2}-2.1x_{1}^{4}+\frac{1}{3}x_{1}^{6}+x_{1}x_{2}-4x_{2}^{2}+4x_{2}^{4}$	2	[−5,5]	−1.0316
$f_{15}(x)=-\frac{1+\cos(12\sqrt{x_{1}^{2}+x_{2}^{2}})}{0.5(x_{1}^{2}+x_{2}^{2})+2}$	2	[−5.12,5.12]	−1
$\begin{array}{l} f_{16}(x)=[1+{\left(x_{1}+x_{2}+1\right)}^{2}\left(19-14x_{1}+3x_{1}^{2}-14x_{2}+6x_{1}x_{2}+3x_{2}^{2}\right)]\\ \times [30+{\left(2x_{1}-3x_{2}\right)}^{2}(18-32x_{1}+12x_{1}^{2}+48x_{2}-36x_{1}x_{2}+27x_{2}^{2})] \end{array}$	2	[−2,2]	3
$f_{17}(x)=-\sum_{i=1}^{4}c_{i}\exp(-\sum_{j=1}^{6}a_{ij}{\left(x_{j}-p_{ij}\right)}^{2})$	6	[0, 1]	−3.32
$f_{18}(x)=-\sum_{i=1}^{5}{\left[(x-a_{i}){\left(x-a_{i}\right)}^{T}+c_{i}\right]}^{-1}$	4	[0, 10]	−10.1532
$f_{19}(x)=-\sum_{i=1}^{7}{\left[(x-a_{i}){\left(x-a_{i}\right)}^{T}+c_{i}\right]}^{-1}$	4	[0,10]	−10.4029
$f_{20}(x)=-\sum_{i=1}^{10}{\left[(x-a_{i}){\left(x-a_{i}\right)}^{T}+c_{i}\right]}^{-1}$	4	[0,10]	−10.5364
$f_{21}(x)=-\cos(x_{1})\cos(x_{2})\exp(-{\left(x_{1}-\pi \right)}^{2}-{\left(x_{2}-\pi \right)}^{2})$	2	[−2π,2π]	−1
$f_{22}(x)=0.5+\frac{\sin^{2}\left(\sqrt{x_{1}^{2}+x_{2}^{2}}\right)-0.5}{{\left(1+0.001(x_{1}^{2}+x_{2}^{2})\right)}^{2}}$	2	[−100,100]	−1
$f_{23}(x)=\sum_{i=1}^{n}x_{i}\sin(x_{i})+0.1x_{i}$	10	[−10,10]	0

**Table 3:** Comparative performance results of benchmark test functions.

Function	Result	LPSO	SBKA	ESGJO	RLWOA	MSSCSO	PO	NRBO	EGO	AO	FATA	HEOA	GCRA	TLGCRA
$f_{1}$	Best	6.17 × 10^−28^	0	0	0	0	0	0	0	5.86 × 10^−51^	0	1.7 × 10^−193^	0	0
	Worst	4.56 × 10^−16^	2.2 × 10^−284^	0	0	0	0	0	0	1.26 × 10^−36^	0	4.1 × 10^−163^	0	0
	Mean	2.60 × 10^−17^	7.5 × 10^−286^	0	0	0	0	0	0	7.51 × 10^−38^	0	1.7 × 10^−164^	0	0
	Std	9.39 × 10^−17^	0	0	0	0	0	0	0	2.62 × 10^−37^	0	0	0	0
$f_{2}$	Best	4.87 × 10^−17^	1.8 × 10^−216^	0	1.5 × 10^−261^	5.6 × 10^−217^	3.4 × 10^−275^	0	0	1.41 × 10^−36^	0	7.49 × 10^−92^	0	0
	Worst	2.47 × 10^−8^	1.2 × 10^−151^	3.6 × 10^−283^	7.8 × 10^−223^	3.2 × 10^−186^	6.8 × 10^−267^	4.2 × 10^−306^	0	8.28 × 10^−26^	0	4.25 × 10^−75^	0	0
	Mean	8.90 × 10^−10^	3.9 × 10^−153^	1.2 × 10^−284^	2.8 × 10^−224^	1.1 × 10^−187^	4.1 × 10^−268^	1.5 × 10^−307^	0	7.50 × 10^−27^	0	1.42 × 10^−76^	0	0
	Std	4.51 × 10^−9^	2.1 × 10^−152^	0	0	0	0	0	0	1.82 × 10^−26^	0	7.75 × 10^−76^	0	0
$f_{3}$	Best	6.16 × 10^−18^	0	0	0	1.9 × 10^−280^	0	0	0	3.60 × 10^−17^	0	7.5 × 10^−217^	0	0
	Worst	3.69 × 10^−11^	5.8 × 10^−302^	0	0	3.1 × 10^−232^	0	0	0	0.000685	0	4.4 × 10^−207^	0	0
	Mean	2.66 × 10^−12^	1.9 × 10^−303^	0	0	1.6 × 10^−233^	0	0	0	5.04 × 10^−5^	0	1.9 × 10^−208^	0	0
	Std	8.34 × 10^−12^	0	0	0	0	0	0	0	0.000139	0	0	0	0
$f_{4}$	Best	4.48 × 10^−13^	8.6 × 10^−211^	4.54 × 10^−97^	3.2 × 10^−250^	0	3.1 × 10^−277^	0	0	3.46 × 10^−7^	0	9.35 × 10^−61^	0	0
	Worst	4.67 × 10^−6^	4.5 × 10^−186^	1.60 × 10^−79^	2.4 × 10^−200^	0	5.6 × 10^−267^	8.0 × 10^−299^	0	7.22 × 10^−5^	0	1.54 × 10^−47^	0	0
	Mean	1.59 × 10^−7^	1.6 × 10^−187^	5.34 × 10^−81^	1.5 × 10^−201^	0	1.9 × 10^−268^	2.9 × 10^−300^	0	2.44 × 10^−5^	0	5.39 × 10^−49^	0	0
	Std	8.53 × 10^−7^	0	2.92 × 10^−80^	0	0	0	0	0	1.87 × 10^−5^	0	2.80 × 10^−48^	0	0
$f_{5}$	Best	26.99573	23.00674	4.224882	26.13592	24.23888	0.000184	26.32771	26.01045	26.33154	0.005311	0.158756	1.57 × 10^−10^	9.45 × 10^−10^
	Worst	28.86971	28.88965	26.26634	27.96951	26.25227	25.37424	28.84175	28.72581	26.80314	28.77661	2.548551	4.36 × 10^−5^	9.62 × 10^−6^
	Mean	27.65382	25.37627	23.48965	26.79924	25.37698	23.93754	27.33898	27.22030	26.60947	7.866659	0.666097	5.01 × 10^−6^	6.23 × 10^−7^
	Std	0.661124	1.497455	4.975964	0.493509	0.519968	4.536083	0.728929	0.699095	0.126309	10.24118	0.469452	1.02 × 10^−5^	1.75 × 10^−6^
$f_{6}$	Best	2.323389	9.78 × 10^−6^	1.38 × 10^−5^	0.001227	2.03 × 10^−5^	6.23 × 10^−11^	0.894278	3.378836	2.35 × 10^−10^	0.000112	0.000215	3.61 × 10^−12^	6.97 × 10^−15^
	Worst	4.238404	3.252882	0.000160	0.221730	5.30 × 10^−5^	4.38 × 10^−9^	3.160329	4.841181	5.58 × 10^−5^	2.762312	0.643056	7.37 × 10^−7^	1.51 × 10^−8^
	Mean	3.379689	0.413212	9.66 × 10^−5^	0.023539	3.84 × 10^−5^	1.24 × 10^−9^	2.188924	4.064612	5.18 × 10^−6^	0.382000	0.165559	9.95 × 10^−8^	2.16 × 10^−9^
	Std	0.427587	0.654476	3.74 × 10^−5^	0.057471	8.52 × 10^−6^	1.07 × 10^−9^	0.450553	0.373507	1.11 × 10^−5^	0.788705	0.185923	1.80 × 10^−7^	3.96 × 10^−9^
$f_{7}$	Best	0.095865	8.38 × 10^−6^	2.73 × 10^−6^	1.58 × 10^−6^	2.45 × 10^−8^	7.18 × 10^−7^	1.58 × 10^−6^	9.26 × 10^−7^	0.000187	8.93 × 10^−7^	2.97 × 10^−6^	5.43 × 10^−6^	1.34 × 10^−8^
	Worst	1.142904	0.000172	0.000261	0.000156	0.000810	0.000212	0.000223	4.93 × 10^−5^	0.002812	9.41 × 10^−5^	0.00021	0.000137	1.30 × 10^−6^
	Mean	0.607646	7.93 × 10^−5^	0.000106	4.42 × 10^−5^	0.000183	6.54 × 10^−5^	6.87 × 10^−5^	1.20 × 10^−5^	0.001062	2.23 × 10^−5^	6.63 × 10^−5^	4.18 × 10^−5^	3.38 × 10^−7^
	Std	0.334056	5.57 × 10^−5^	7.03 × 10^−5^	3.88 × 10^−5^	0.000190	6.14 × 10^−5^	5.74 × 10^−5^	1.02 × 10^−5^	0.000603	2.31 × 10^−5^	5.67 × 10^−5^	3.28 × 10^−5^	3.36 × 10^−7^
$f_{8}$	Best	0	0	0	0	0	0	0	0	0	0	29.86209	0	0
	Worst	0	0	0	0	0	0	0	0	0	0	40.91937	0	0
	Mean	0	0	0	0	0	0	0	0	0	0	30.60579	0	0
	Std	0	0	0	0	0	0	0	0	0	0	2.483664	0	0
$f_{9}$	Best	4.00 × 10^−15^	4.44 × 10^−16^	4.44 × 10^−16^	4.44 × 10^−16^	4.44 × 10^−16^	4.44 × 10^−16^	4.44 × 10^−16^	4.44 × 10^−16^	4.00 × 10^−15^	4.44 × 10^−16^	4.44 × 10^−16^	4.44 × 10^−16^	4.44 × 10^−16^
	Worst	2.54 × 10^−8^	4.44 × 10^−16^	4.44 × 10^−16^	4.44 × 10^−16^	4.44 × 10^−16^	4.44 × 10^−16^	4.44 × 10^−16^	4.44 × 10^−16^	1.47 × 10^−14^	4.44 × 10^−16^	4.44 × 10^−16^	4.44 × 10^−16^	4.44 × 10^−16^
	Mean	1.73 × 10^−9^	4.44 × 10^−16^	4.44 × 10^−16^	4.44 × 10^−16^	4.44 × 10^−16^	4.44 × 10^−16^	4.44 × 10^−16^	4.44 × 10^−16^	8.85 × 10^−15^	4.44 × 10^−16^	4.44 × 10^−16^	4.44 × 10^−16^	4.44 × 10^−16^
	Std	5.79 × 10^−9^	0	0	0	0	0	0	0	3.02 × 10^−15^	0	0	0	0
$f_{10}$	Best	0	0	0	0	0	0	0	0	0	0	0	0	0
	Worst	8.88 × 10^−16^	0	0	0	0	0	0	0	0.075727	0	0.101722	0	0
	Mean	2.96 × 10^−17^	0	0	0	0	0	0	0	0.003756	0	0.023340	0	0
	Std	1.62 × 10^−16^	0	0	0	0	0	0	0	0.014409	0	0.029233	0	0
$f_{11}$	Best	0.233942	0.006725	1.56 × 10^−6^	0.000412	1.83 × 10^−6^	1.08 × 10^−11^	0.089115	0.242309	3.48 × 10^−13^	4.54 × 10^−7^	0.001094	1.52 × 10^−12^	1.70 × 10^−17^
	Worst	0.480233	0.243470	1.09 × 10^−5^	0.013227	5.43 × 10^−6^	4.42 × 10^−10^	0.252648	0.514559	9.63 × 10^−7^	0.012460	0.704513	2.16 × 10^−8^	2.43 × 10^−9^
	Mean	0.351792	0.054152	5.78 × 10^−6^	0.003329	3.28 × 10^−6^	8.59 × 10^−11^	0.173465	0.363572	6.23 × 10^−8^	0.001152	0.158998	1.89 × 10^−9^	3.17 × 10^−10^
	Std	0.062871	0.065641	2.49 × 10^−6^	0.003566	9.18 × 10^−7^	8.63 × 10^−11^	0.043237	0.063102	1.79 × 10^−7^	0.002656	0.171394	3.98 × 10^−9^	5.95 × 10^−10^
$f_{12}$	Best	1.825741	0.306594	1.19 × 10^−5^	0.007623	1.70 × 10^−5^	7.57 × 10^−11^	1.346936	0.157345	2.41 × 10^−12^	0.000121	0.000122	1.10 × 10^−10^	2.26 × 10^−15^
	Worst	2.404141	2.577837	0.000202	0.307367	7.04 × 10^−5^	3.79 × 10^−9^	2.978854	1.233280	0.586973	0.059365	0.008389	1.48 × 10^−7^	5.94 × 10^−8^
	Mean	2.058088	1.070782	8.74 × 10^−5^	0.042714	4.48 × 10^−5^	9.06 × 10^−10^	2.002126	0.526782	0.111098	0.008646	0.001567	2.8 × 10^−8^	3.58 × 10^−9^
	Std	0.153843	0.541230	4.96 × 10^−5^	0.054478	1.24 × 10^−5^	8.44 × 10^−10^	0.436751	0.261932	0.133287	0.014801	0.001719	3.82 × 10^−8^	1.08 × 10^−8^
$f_{13}$	Best	0.998004	0.998004	0.998004	0.998004	0.998004	0.998004	0.998004	0.998004	0.998004	0.998004	0.998004	0.998004	0.998004
	Worst	2.982105	0.998004	0.998004	2.982105	0.998004	0.998004	12.67051	1.022900	10.76318	12.67051	12.67051	0.998004	0.998004
	Mean	1.262640	0.998004	0.998004	1.196414	0.998004	0.998004	2.567193	0.999882	1.522078	1.776171	8.196286	0.998004	0.998004
	Std	0.685959	1.17 × 10^−16^	3.91 × 10^−11^	0.605406	7.85 × 10^−12^	0	3.132628	0.005168	1.828591	2.961409	4.666077	1.91 × 10^−12^	2.08 × 10^−10^
$f_{14}$	Best	−1.03163	−1.03163	−1.03163	−1.03163	−1.03163	−1.03163	−1.03163	−1.03163	−1.03163	−1.03163	−1.03163	−1.03086	−1.03163
	Worst	−1.03159	−1.03163	−1.03157	−1.03160	−1.03163	−1.03163	−1.03163	−1.03044	−1.03141	−1.03163	−1.03140	−0.16669	−1.03132
	Mean	−1.03161	−1.03163	−1.03162	−1.03162	−1.03163	−1.03163	−1.03163	−1.03133	−1.03161	−1.03163	−1.03159	−0.92899	−1.03156
	Std	1.18 × 10^−5^	6.45 × 10^−16^	1.17 × 10^−5^	7.32 × 10^−6^	1.84 × 10^−8^	6.65 × 10^−16^	5.61 × 10^−16^	0.000279	5.39 × 10^−5^	2.02 × 10^−8^	5.50 × 10^−5^	0.186879	7.06 × 10^−5^
$f_{15}$	Best	−1	−1	−1	−1	−1	−1	−1	−1	−1	−1	−1	−1	−1
	Worst	−1	−1	−1	−1	−1	−0.93625	−1	−1	−1	−1	−1	−1	−1
	Mean	−1	−1	−1	−1	−1	−0.99787	−1	−1	−1	−1	−1	−1	−1
	Std	0	0	0	0	0	0.011640	0	0	0	0	0	9.56 × 10^−11^	0
$f_{16}$	Best	3	3	3	3	3	3	3	3	3	3	3.000023	3.155245	3
	Worst	3.000020	3	3.000005	3.000398	3.000001	3	3	3.000775	3.044026	3.000002	5.038619	32.68453	3
	Mean	3.000003	3	3.000001	3.000035	3	3	3	3.000096	3.003641	3	3.077565	20.75731	3
	Std	3.94 × 10^−6^	8.45 × 10^−16^	9.77 × 10^−7^	7.49 × 10^−5^	2.92 × 10^−7^	1.60 × 10^−15^	2.08 × 10^−15^	0.000162	0.008607	4.38 × 10^−7^	0.371076	10.80034	1.29 × 10^−15^
$f_{17}$	Best	−3.24827	−3.32200	−3.32187	−3.32188	−3.32198	−3.32200	−3.32200	−3.32067	−3.32200	−3.31234	−3.31109	−3.02968	−3.32200
	Worst	−2.60793	−3.08668	−2.84044	−3.01551	−3.08097	−3.20310	−3.12738	−3.06527	−3.20166	−3.15602	−3.07874	−1.16984	−3.32200
	Mean	−3.07156	−3.24356	−3.17729	−3.22125	−3.19381	−3.26651	−3.25362	−3.25127	−3.27040	−3.22902	−3.19323	−1.90937	−3.32200
	Std	0.122112	0.080579	0.093233	0.111599	0.058134	0.060328	0.067066	0.090955	0.060001	0.054439	0.073701	0.482049	1.39 × 10^−15^
$f_{18}$	Best	−5.02293	−10.1532	−10.1488	−10.1531	−10.1520	−10.1532	−10.1532	−9.68488	−10.1532	−10.1532	−10.1497	−10.1532	−10.1532
	Worst	−4.43574	−2.63047	−4.98866	−10.1353	−5.05501	−10.1532	−2.63047	−5.00653	−2.62952	−10.1531	−5.05492	−10.1466	−10.1527
	Mean	−4.77743	−9.65168	−6.23529	−10.1481	−6.41305	−10.1532	−9.70229	−5.60537	−5.85794	−10.1532	−8.57784	−10.1528	−10.1531
	Std	0.135751	1.908577	2.181703	0.004464	2.290323	6.85 × 10^−15^	1.630944	1.472010	2.813123	3.65 × 10^−5^	1.226612	0.001183	9.90 × 10^−5^
$f_{19}$	Best	−7.50047	−10.4029	−10.4004	−10.4024	−10.4009	−10.4029	−10.4029	−10.3094	−10.4029	−10.4029	−10.4014	−10.4028	−10.4029
	Worst	−3.67892	−2.76590	−5.08609	−10.3765	−5.08740	−3.72430	−2.76590	−5.03846	−2.74761	−10.4026	−5.34575	−10.3992	−10.4025
	Mean	−4.88800	−9.92575	−6.32302	−10.3964	−6.14970	−10.1803	−9.80923	−5.57185	−6.89784	−10.4028	−9.36166	−10.4024	−10.4028
	Std	0.712924	1.820358	2.276045	0.005228	2.160514	1.219347	1.783241	1.519435	3.61054	4.66 × 10^−5^	1.273898	0.000800	9.14 × 10^−5^
$f_{20}$	Best	−6.93263	−10.5364	−10.5352	−10.5360	−10.5363	−10.5364	−10.5364	−10.0387	−10.5364	−10.5363	−10.5340	−10.5363	−10.5363
	Worst	−3.65298	−2.42173	−5.12748	−10.5232	−5.12801	−10.5239	−6.61995	−5.09156	−2.42144	−10.5355	−5.60674	−10.5303	−10.5356
	Mean	−4.90956	−9.54870	−8.90112	−10.5325	−6.92906	−10.5360	−10.2513	−5.28140	−6.70094	−10.5362	−9.56300	−10.5357	−10.5362
	Std	0.577578	2.574645	2.512312	0.003327	2.590079	0.002277	0.834435	0.898551	3.740067	0.000159	1.193983	0.001264	0.000127
$f_{21}$	Best	−0.99999	−1	−1	−1	−1	−1	−1	−1	−1	−1	−1	−1	−1
	Worst	−0.99750	−1	−0.99998	−1	−1	−1	−1	−0.99958	−0.99901	−1	−0.99992	−1	−1
	Mean	−0.99946	−1	−1	−1	−1	−1	−1	−0.99988	−0.99992	−1	−0.99999	−1	−1
	Std	0.000574	0	4.11 × 10^−6^	1.06 × 10^−6^	1.06 × 10^−6^	0	0	0.000114	0.000223	2.08 × 10^−7^	1.68 × 10^−5^	2.89 × 10^−7^	2.85 × 10^−7^
$f_{22}$	Best	−1	−1	−1	−1	−1	−1	−1	−1	−1	−1	−1	−1	−1
	Worst	−1	−1	−1	−1	−1	−0.99028	−1	−1	−1	−1	−0.99028	−1	−1
	Mean	−1	−1	−1	−1	−1	−0.99935	−1	−1	−1	−1	−0.99676	−1	−1
	Std	0	0	0	0	0	0.002465	0	0	0	0	0.004658	1.67 × 10^−10^	0
$f_{23}$	Best	3.11 × 10^−22^	2.2 × 10^−213^	0	1.2 × 10^−276^	9.5 × 10^−302^	2.0 × 10^−275^	0	0	1.17 × 10^−87^	0	5.79 × 10^−98^	1.45 × 10^−13^	0
	Worst	4.04 × 10^−15^	4.5 × 10^−158^	3.9 × 10^−253^	1.3 × 10^−216^	9.0 × 10^−263^	4.1 × 10^−266^	0	0	9.34 × 10^−51^	0.000421	0.272130	5.48 × 10^−5^	0
	Mean	2.99 × 10^−16^	1.5 × 10^−159^	1.3 × 10^−254^	4.5 × 10^−218^	3.0 × 10^−264^	1.6 × 10^−267^	0	0	3.19 × 10^−52^	2.78 × 10^−5^	0.023033	3.08 × 10^−6^	0
	Std	8.15 × 10^−16^	8.2 × 10^−159^	0	0	0	0	0	0	1.70 × 10^−51^	0.000106	0.072251	1.02 × 10^−5^	0

**Table 4:** Contrastive results of the *p*-value Wilcoxon rank-sum test on the benchmark functions.

Function	TLGCRA vs. LPSO	TLGCRA vs. SBKA	TLGCRA vs. ESGJO	TLGCRA vs. RLWOA	TLGCRA vs. MSSCSO	TLGCRA vs. PO	TLGCRA vs. NRBO	TLGCRA vs. EGO	TLGCRA vs. AO	TLGCRA vs. FATA	TLGCRA vs. HEOA	TLGCRA vs. GCRA
$f_{1}$	1.21 × 10^−12^	0.333711	N/A	N/A	N/A	N/A	N/A	N/A	1.21 × 10^−12^	N/A	1.21 × 10^−12^	N/A
$f_{2}$	1.21 × 10^−12^	1.21 × 10^−12^	1.93 × 10^−10^	1.21 × 10^−12^	1.21 × 10^−12^	1.21 × 10^−12^	4.19 × 10^−2^	N/A	1.21 × 10^−12^	N/A	1.21 × 10^−12^	N/A
$f_{3}$	1.21 × 10^−12^	0.160802	N/A	N/A	1.21 × 10^−12^	N/A	N/A	N/A	1.21 × 10^−12^	N/A	1.21 × 10^−12^	N/A
$f_{4}$	1.21 × 10^−12^	1.21 × 10^−12^	1.21 × 10^−12^	1.21 × 10^−12^	N/A	1.21 × 10^−12^	4.57 × 10^−12^	N/A	1.21 × 10^−12^	N/A	1.21 × 10^−12^	N/A
$f_{5}$	3.02 × 10^−11^	3.02 × 10^−11^	3.02 × 10^−11^	3.02 × 10^−11^	3.02 × 10^−11^	3.02 × 10^−11^	3.02 × 10^−11^	3.02 × 10^−11^	3.02 × 10^−11^	3.02 × 10^−11^	3.02 × 10^−11^	2.38 × 10^−3^
$f_{6}$	3.02 × 10^−11^	3.02 × 10^−11^	3.02 × 10^−11^	3.02 × 10^−11^	3.02 × 10^−11^	1.22 × 10^−2^	3.02 × 10^−11^	3.02 × 10^−11^	9.76 × 10^−10^	3.02 × 10^−11^	3.02 × 10^−11^	2.00 × 10^−5^
$f_{7}$	3.02 × 10^−11^	3.02 × 10^−11^	3.02 × 10^−11^	3.02 × 10^−11^	4.62 × 10^−10^	4.50 × 10^−11^	3.02 × 10^−11^	4.50 × 10^−11^	3.02 × 10^−11^	4.50 × 10^−11^	3.02 × 10^−11^	3.02 × 10^−11^
$f_{8}$	N/A	N/A	N/A	N/A	N/A	N/A	N/A	N/A	N/A	N/A	1.21 × 10^−12^	N/A
$f_{9}$	1.21 × 10^−12^	N/A	N/A	N/A	N/A	N/A	N/A	N/A	2.01 × 10^−13^	N/A	N/A	N/A
$f_{10}$	0.333711	N/A	N/A	N/A	N/A	N/A	N/A	N/A	8.15 × 10^−1^	N/A	5.37 × 10^−6^	N/A
$f_{11}$	3.02 × 10^−11^	3.02 × 10^−11^	3.02 × 10^−11^	3.02 × 10^−11^	3.02 × 10^−11^	9.70 × 10^−1^	3.02 × 10^−11^	3.02 × 10^−11^	2.25 × 10^−4^	3.02 × 10^−11^	3.02 × 10^−11^	3.18 × 10^−3^
$f_{12}$	3.02 × 10^−11^	3.02 × 10^−11^	3.02 × 10^−11^	3.02 × 10^−11^	3.02 × 10^−11^	3.25 × 10^−2^	3.02 × 10^−11^	3.02 × 10^−11^	1.20 × 10^−8^	3.02 × 10^−11^	3.02 × 10^−11^	1.64 × 10^−5^
$f_{13}$	3.02 × 10^−11^	5.18 × 10^−12^	0.641424	5.86 × 10^−6^	0.017649	1.21 × 10^−12^	7.64 × 10^−2^	3.02 × 10^−11^	1.08 × 10^−2^	5.82 × 10^−3^	4.98 × 10^−11^	8.70 × 10^−8^
$f_{14}$	6.77 × 10^−5^	5.14 × 10^−12^	1.1 × 10^−8^	1.73 × 10^−6^	3.02 × 10^−11^	2.36 × 10^−12^	1.41 × 10^−11^	2.43 × 10^−5^	3.70 × 10^−7^	3.02 × 10^−11^	1.56 × 10^−2^	3.02 × 10^−11^
$f_{15}$	N/A	N/A	N/A	N/A	N/A	3.33 × 10^−2^	N/A	N/A	N/A	N/A	N/A	4.79 × 10^−8^
$f_{16}$	3.16 × 10^−12^	7.1 × 10^−5^	3.16 × 10^−12^	3.16 × 10^−12^	3.16 × 10^−12^	6.33 × 10^−4^	2.84 × 10^−4^	3.16 × 10^−12^	1.02 × 10^−10^	3.16 × 10^−12^	3.16 × 10^−12^	3.16 × 10^−12^
$f_{17}$	2.36 × 10^−12^	2.36 × 10^−12^	2.36 × 10^−12^	2.36 × 10^−12^	2.36 × 10^−12^	7.46 × 10^−4^	2.36 × 10^−12^	2.36 × 10^−12^	7.43 × 10^−10^	2.36 × 10^−12^	2.36 × 10^−12^	2.36 × 10^−12^
$f_{18}$	3.02 × 10^−11^	4.36 × 10^−9^	3.02 × 10^−11^	4.98 × 10^−11^	3.02 × 10^−11^	7.57 × 10^−12^	2.05 × 10^−4^	3.02 × 10^−11^	4.03 × 10^−5^	4.73 × 10^−2^	3.02 × 10^−11^	5.26 × 10^−4^
$f_{19}$	3.02 × 10^−11^	6.27 × 10^−9^	3.02 × 10^−11^	3.02 × 10^−11^	3.02 × 10^−11^	7.75 × 10^−11^	8.16 × 10^−2^	3.02 × 10^−11^	6.51 × 10^−1^	5.36 × 10^−2^	3.02 × 10^−11^	7.95 × 10^−3^
$f_{20}$	3.02 × 10^−11^	8.55 × 10^−7^	3.02 × 10^−11^	4.08 × 10^−11^	2.15 × 10^−10^	2.84 × 10^−10^	5.77 × 10^−3^	3.02 × 10^−11^	7.72 × 10^−2^	1.15 × 10^−2^	3.02 × 10^−11^	1.71 × 10^−2^
$f_{21}$	3.02 × 10^−11^	1.21 × 10^−12^	1.09 × 10^−10^	4.42 × 10^−6^	0.000691	1.21 × 10^−12^	1.21 × 10^−12^	3.02 × 10^−11^	7.64 × 10^−3^	2.17 × 10^−2^	5.19 × 10^−7^	9.94 × 10^−1^
$f_{22}$	N/A	N/A	N/A	N/A	N/A	1.60 × 10^−2^	N/A	N/A	N/A	N/A	1.38 × 10^−4^	5.84 × 10^−9^
$f_{23}$	1.21 × 10^−12^	1.21 × 10^−12^	0.333711	1.21 × 10^−12^	1.21 × 10^−12^	1.21 × 10^−12^	N/A	N/A	1.21 × 10^−12^	8.15 × 10^−2^	1.21 × 10^−12^	1.21 × 10^−12^

**Table 5:** Comparative outcomes of three-bar truss engineering design task.

Algorithm	Optimum Variables		Optimum Weight
	$A_{1}$	$A_{2}$	
ESOA [41]	0.788192	0.409618	263.896
DE [42]	0.788675	0.408248	263.896
HGS [43]	0.7884562	0.40886831	263.8959
I-GWO [44]	0.784408	0.420579	263.9220
HOA [45]	0.78192	0.4268	263.8841
SELO [46]	0.7878	0.4108	263.8964
HBO [46]	0.7887	0.4082	263.8959
LFD [46]	0.7879	0.4106	263.8963
SETO [46]	0.7886	0.4083	263.8958
ARSCA [47]	0.7887	0.4081	263.8958
CPO [47]	0.7885	0.4088	263.8959
TLGCRA	0.78699	0.41384	263.8786

**Table 6:** Comparative outcomes of the tubular column engineering design task.

Algorithm	Optimum Variables		Optimum Cost
	d	t	
CS [48]	5.45139	0.29196	26.53217
ISA [49]	5.45115623	0.29196547	26.5313
Hsu and Liu [50]	5.4507	0.292	25.5316
FLA [51]	5.4801	0.2905	26.563266
GSA-GA [52]	5.45115623	0.29196548	26.531328
AGQPSO [53]	5.451156	0.29196	26.531328
KH [54]	5.451278	0.291957	26.5314
EM [55]	5.452383	0.29190	26.53380
HEM [55]	5.451083	0.29199	26.53227
AOS [56]	N/A	N/A	26.5313783
GSA [57]	5.451163397	0.291965509	26.531364472
TLGCRA	5.4637	0.29133	26.5263

**Table 7:** Comparative outcomes of the speed reducer engineering design task.

Algorithm	Optimum Variables							Optimum Weight
	*b*	*m*	*z*	*l*_1_	*l*_2_	*d*_1_	*d*_2_	
CS [58]	3.5015	0.7	17	7.605	7.8181	3.352	5.2875	3000.981
ABC [59]	3.5	0.7	17	7.3	7.8	3.35022	5.28668	2997.05841
MVO [60]	3.508502	0.7	17	7.392843	7.816034	3.358073	5.286777	3002.928
AAO [61]	3.499	0.6999	17	7.3	7.8	3.3502	5.2877	2997.058
MFPA [62]	3.5	0.7	17	7.3	7.8005	3.35021	5.28668	2996.219
HHO [63]	3.4965	0.7	17	7.3	7.8	3.3519	5.2856	2997.10
CSO [64]	3.48	0.7	17	8.26	7.95	3.34	5.28	2997.7074
STOA [65]	3.50124	0.7	17	7.3	7.8	3.33425	5.26538	2995.9758
RUN [66]	3.507125	0.7	17	7.307812	7.8078	3.356534	5.29705	3004.852
MSA [66]	3.505551	0.7	17	8.308882	7.8079	3.357677	5.29502	3012.079
SBS [67]	3.506122	0.700006	17	7.549126	7.85933	3.365576	5.289773	3008.981
SFOA [68]	3.5	0.7	17	7.3	7.8	3.35	5.287	2996.348
TLGCRA	3.5213	0.7001	17	7.3	7.8013	3.3531	5.2863	2995.4217

**Table 8:** Comparison results of the pressure vessel design problem.

Algorithm	Optimum Variables				Optimum Cost
	$T_{s}$	$T_{h}$	R	L	
CPSO [69]	0.8125	0.4375	42.0913	176.7465	6061.0777
CDE [70]	0.8125	0.4375	42.0984	176.6376	6059.7340
SAP [71]	0.8125	0.4375	40.3239	200.0000	6288.7445
DA [72]	0.782825	0.384649	40.3196	200	5923.11
DE [73]	0.8125	0.4375	42.098446	176.636047	6059.70166
CLPSO [74]	0.7824	0.3867	40.5352	197.2093	5903.5077
SADE [74]	0.7782	0.3847	40.3196	200	5891.5859
FOX [75]	0.780559	0.3860622	40.43913	198.5199	5894.5033
ETO [76]	0.7865062	0.3917354	40.54479	199.1287	5984.8509
BBOA [76]	0.8125	0.4375	42.098456	176.62446	6059.714
GRO [77]	0.7787153	0.384967	40.347943	199.6061	5886.4068
SFOA [78]	0.77817	0.38465	40.31962	200	5885.333
TLGCRA	0.7463	0.3631	41.3014	200	5884.2153

**Table 9:** Comparative outcomes of the gear train engineering design task.

Algorithm	Optimum Variables				Optimum Cost
	$n_{A}$	$n_{B}$	$n_{C}$	$n_{D}$	
GJO [79]	56.7899	18.5239	19.8299	44.8316	1.7754 × 10^−19^
GTO [80]	34.65788	12	12	28.79761	2.42 × 10^−18^
BWO [81]	50	18	17	46	7.5421 × 10^−17^
GTBO [81]	51	32	12	52	2.0211 × 10^−20^
MPA [82]	38.7588	14.6570	12.0713	31.6393	1.6923 × 10^−23^
SOA [82]	58.2626	12	42.0304	60	6.2215 × 10^−16^
STOA [82]	60	24.6745	20.9436	59.696	7.0004 × 10^−15^
TSA [82]	54.0750	18.1623	20.5854	47.9214	5.8199 × 10^−16^
CPO [82]	58.3836	42.0934	12.0060	59.9955	4.1837 × 10^−25^
SO [83]	56.6	21.6	22.7	59.9	6.57 × 10^−15^
RIME [83]	59.2	16.7	25.2	49	5.0 × 10^−15^
DBO [83]	52.5	31.3	14.5	60	3.67 × 10^−26^
TLGCRA	52	30	15	59	4.5921 × 10^−14^

**Table 10:** Comparative outcomes of the multi-disk clutch brake engineering design task.

Algorithm	Optimum Variables					Optimum Weight
	$r_{i}$	$r_{0}$	t	F	Z	
MFO [84]	70	90	1	910	3	0.313656
PVS [85]	70	90	1	980	3	0.31366
RSA [86]	70.0347	90.0349	1	801.7285	2.9740	0.31176
QSMFO [87]	80	101.3002	3	600	9	0.2902
EPO [88]	70	90	1.5	1000	3	0.4704
SOA [88]	77.1459	97.2218	1	628.1937	3.3809	0.3758
TACPSO [88]	75.0044	95.0044	1	1000	2.1779	0.2648
MPSO [88]	70	90	1	1000	2.3128	0.2598
GTO [89]	69.99975	90	1	1000	2	0.23525
WOA [89]	69.8921	90	1	79.4724	2	0.23635
COA [89]	69.99622	90	1	1000	2	0.23528
SO [89]	69.99642	90	1	984	2	0.23528
TLGCRA	70	90	1	958	2	0.2351

## Data Availability

The data presented in this study are available on request from the corresponding author. The MATLAB code developed for this study is available from the corresponding author upon reasonable request.
